# Loss of CBY1 results in a ciliopathy characterized by features of Joubert syndrome

**DOI:** 10.1002/humu.24127

**Published:** 2020-11-01

**Authors:** Daniel Epting, Lokuliyange D. S. Senaratne, Elisabeth Ott, Asbjørn Holmgren, Dulika Sumathipala, Selma M. Larsen, Julia Wallmeier, Diana Bracht, Kari‐Anne M. Frikstad, Suzanne Crowley, Alma Sikiric, Tuva Barøy, Barbara Käsmann‐Kellner, Eva Decker, Christian Decker, Nadine Bachmann, Sebastian Patzke, Ian G. Phelps, Nicholas Katsanis, Rachel Giles, Miriam Schmidts, Manuela Zucknick, Soeren S. Lienkamp, Heymut Omran, Erica E. Davis, Dan Doherty, Petter Strømme, Eirik Frengen, Carsten Bergmann, Doriana Misceo

**Affiliations:** ^1^ Department of Medicine IV, Faculty of Medicine Medical Center‐University of Freiburg Freiburg Germany; ^2^ Department of Medical Genetics Oslo University Hospital, University of Oslo Oslo Norway; ^3^ Division of Pediatric and Adolescent Medicine Oslo University Hospital, University of Oslo Oslo Norway; ^4^ Klinik für Kinder‐ und Jugendmedizin Universitätsklinikum Münster Münster Germany; ^5^ Department of Radiation Biology, Division of Cancer Medicine, Surgery and Transplantation, Institute for Cancer Research Oslo University Hospitals–Norwegian Radium Hospital Oslo Norway; ^6^ Department of Neurohabilitation Oslo University Hospital Oslo Norway; ^7^ Section of Pediatric Ophthalmology and Low Vision, Department of Ophthalmology University of Saarland Homburg Germany; ^8^ Medizinische Genetik Mainz Limbach Genetics Mainz Germany; ^9^ Department of Pediatrics, Seattle Children's Research Institute University of Washington Seattle Washington USA; ^10^ Center for Human Disease Modeling Duke University Medical Center Durham North Carolina USA; ^11^ Department of Nephrology and Hypertension University Medical Center Utrecht Utrecht The Netherlands; ^12^ International Radboud Institute for Molecular Life Sciences Radboud University Nijmegen Nijmegen The Netherlands; ^13^ Oslo Centre for Biostatistics and Epidemiology, Institute for Basic Medical Sciences University of Oslo Oslo Norway; ^14^ Institute of Anatomy University of Zurich Zurich Switzerland

**Keywords:** CBY1, ciliopathy, Joubert syndrome, primary cilia defect, whole exome sequencing, zebrafish

## Abstract

Ciliopathies are clinically and genetically heterogeneous diseases. We studied three patients from two independent families presenting with features of Joubert syndrome: abnormal breathing pattern during infancy, developmental delay/intellectual disability, cerebellar ataxia, molar tooth sign on magnetic resonance imaging scans, and polydactyly. We identified biallelic loss‐of‐function (LOF) variants in *CBY1*, segregating with the clinical features of Joubert syndrome in the families. CBY1 localizes to the distal end of the mother centriole, contributing to the formation and function of cilia. In accordance with the clinical and mutational findings in the affected individuals, we demonstrated that depletion of Cby1 in zebrafish causes ciliopathy‐related phenotypes. Levels of CBY1 transcript were found reduced in the patients compared with controls, suggesting degradation of the mutated transcript through nonsense‐mediated messenger RNA decay. Accordingly, we could detect CBY1 protein in fibroblasts from controls, but not from patients by immunofluorescence. Furthermore, we observed reduced ability to ciliate, increased ciliary length, and reduced levels of the ciliary proteins AHI1 and ARL13B in patient fibroblasts. Our data show that *CBY1* LOF‐variants cause a ciliopathy with features of Joubert syndrome.

## 1 INTRODUCTION

Cilia are structurally and functionally complex organelles that reside on the surface of most cells in the vertebrate body plan. Dysfunctional cilia cause a range of disorders in humans, called ciliopathies, which are clinically and genetically heterogeneous with a wide spectrum of phenotypes (Reiter & Leroux, 2017). Cilia can be divided into two main types: primary cilia that are important signaling hubs and play a role in embryonic life during organogenesis and motile cilia that exert their action in epithelial cells, for example, in the lining of the respiratory tract. Joubert syndrome (JBTS; MIM #213300) is an early onset primary ciliopathy, accompanied by the characteristic mid‐ and hindbrain malformations, including the cerebellar anomaly designated as the “molar tooth sign”, seen in the axial plane on cerebral magnetic resonance imaging (MRI; Romani et al., 2013). Individuals with JBTS present with infantile breathing abnormalities (tachypnea and/or apnea), oculomotor apraxia, cerebellar ataxia, hypotonia, and psychomotor delay (Parisi, 2009). Multiple organ involvement and polydactyly is not unusual. Defective motile cilia, however, as in primary ciliary dyskinesia (PCD; MIM #244400), results in decreased clearance of respiratory tract secretions and is clinically an important differential diagnosis to cystic fibrosis. Individuals with PCD demonstrate chronic oto‐sinopulmonary disease due to mucus retention, and also display other features, which may include heterotaxia and infertility (Zariwala et al., 1993). Despite major ultrastructural similarities between motile and primary cilia, concomitant dysfunction of both organellar subtypes is rarely documented (Horani & Ferkol, 2016; Mitchison & Valente, 2017). Here, we describe three individuals from two unrelated families with biallelic loss‐of‐function (LOF) variants in *CBY1*, presenting with features of JBTS.

## 2 PATIENTS AND METHODS

### 2.1 Ethical considerations

The ethics committees of all relevant participating institutions approved this study. Participant family members signed informed consent for genetic studies and for publication of clinical data. The affected individuals reported in this study were connected through the data‐sharing platform GeneMatcher (Sobreira et al., 2015).

The experiments performed on zebrafish were conducted in accordance with relevant institutional (Regierungspräsidium, Freiburg, Germany) and national guidelines and regulations for animal care and use.

### 2.2 Clinical presentation of the patients

**Figure 1:**
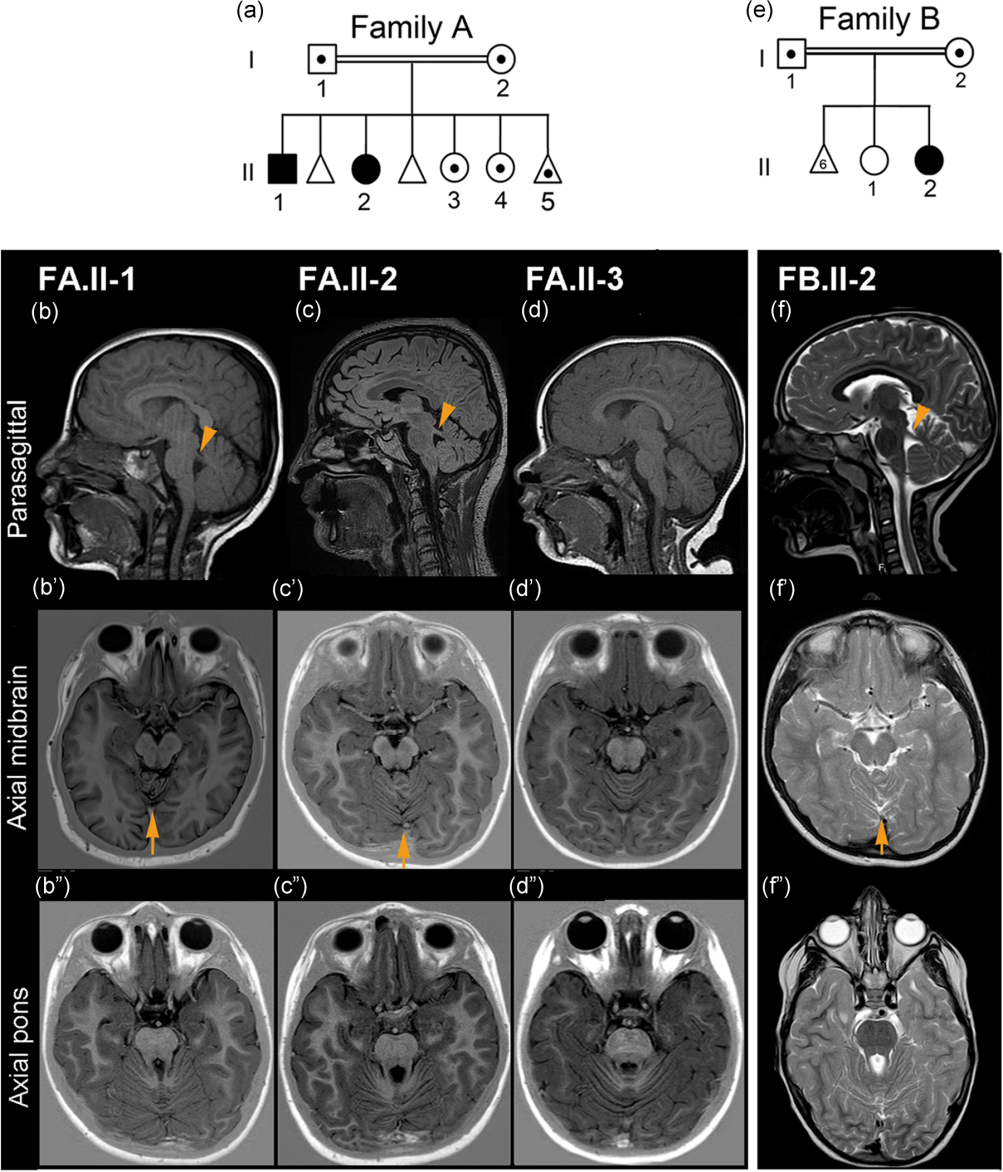
Pedigree of Families A and B and cerebral MRI findings. (a) Pedigree of Family A showing the two affected siblings (black symbols) and carriers (symbols with the dot) of the *CBY1* variant Chr22:g.39067079_39067080del within the family. The first two fetuses were not genotyped. (b−f) Cerebral magnetic resonance imaging examinations in Family A (FA.II‐1, FA.II‐2, FA.II‐3), and Family B (FB.II‐2). In the parasagittal views (b,c,f), the superior cerebellar peduncles (arrowheads) are more horizontally oriented, as opposed to the normal and more vertically oriented peduncle in (d). In the axial midbrain views (bʹ,cʹ,fʹ), cerebellar vermian foliar dysplasia is seen above the arrows. This anomaly is not present in (dʹ). The axial pons views (b″,c″,f″) show elongation of the superior cerebellar peduncles giving a mild “molar tooth” appearance, but not in (d″). Sagittal views (b–d) are T1 and (f) T2 weighted. Axial midbrain and pons views (bʹ–dʹ) and (b″–d″) are inversion recovery and (f–f″) are T2 weighted. (e) Pedigree of Family B showing the affected sibling and her parents, which are carriers of the *CBY1* variant Chr22:g.39064123_39064124dup. FB.II‐1 and the fetuses were not genotyped

In Family A (FA), healthy, first‐cousin parents from Pakistan had a total of seven pregnancies and four living children (Figure 1a). The two elder siblings FA.II‐1 and FA.II‐2 had a clinical diagnosis of JBTS (Table 1). In addition, they displayed organ manifestations likely not attributable to JBTS (Table S1). FA.II‐1 was born at 36 weeks of gestation with birth weight of 2040 g (1 kg < 2.5 percentile). The occipitofrontal head circumference (OFC) at birth was 33 cm (2.5th percentile) and at 5 weeks 34 cm (1 cm < 2.5th percentile); and later OFC measurements stayed at 1 cm < 2.5th percentile. During the first year delayed eye contact and oculomotor apraxia became evident, together with recurrent episodes of tachypnea. Muscular hypotonia was noted from an early age. He walked at 2 years with a wide‐based atactic gait. Cerebral MRI examinations at 1 and 8 years showed elongated and horizontally oriented superior cerebellar peduncles, vermis hypoplasia, and superior cerebellar foliar dysplasia (Figure 1b). He was operated for bilateral calcaneovalgus and pes planus. He was also noted to have agenesis of the lower lateral incisors, small teeth, and fifth finger clinodactyly. At 4 years and 9 months general development was delayed with language skills corresponding to 2.5 years. Speech was impaired due to dysarthria. At 18 years, formal cognitive evaluation performed with the Wechsler Adult Intelligence Scale version IV Full Scale Intelligent Quotient (WAIS‐IV FSIQ) concluded with IQ 64 (confidence interval, 58–69), compatible with mild intellectual disability. Debilitating chronic upper airways symptoms were evident all through his childhood. Chronic serous otitis media led to impaired hearing. He had nasal polyps and chronic rhinitis and sinusitis with persistent retention of mucoid material in the paranasal sinuses (Figure S1). Low nasal nitric oxide (nNO) concentrations were 87 and 47 ppb (normal > 200 ppb), measured twice at 15 years. FA.II‐1 also suffered from a number of other abnormalities and diseases without obvious connection with the JBTS (Table S1), for example, he was operated for late infantile bilateral cataracts between 3 and 4 years, and he needed surgical correction for nasal septum deviation. At 3 years, he was diagnosed with celiac disease and anemia. From 13 years, he suffered subacute episodes of fatigue accompanied by poor appetite and pain in the thoracic cage, neck, and extremities. In parallel with this, serum creatine kinase (CK) concentrations were constantly elevated ranging from 350 to 1200 U/L (reference < 200 U/L) and plasma myoglobin was also elevated with a concentration of 500 ng/ml (reference, 0–85 ng/ml). Neurography and electromyography of upper and lower extremities at 16 and 19 years indicated combined myopathy and axonal (sensory and motor) neuropathy. A precise interpretation was difficult, but myopathy was considered the predominant pathological component. MRI examination of the lower extremities showed infiltration of fat in the muscles and biopsy from his right mid‐gastrocnemius confirmed deposition of fat and connective tissue with extensive myopathic and neurogenic changes. At 21 years, he suffered episodes of nontraumatic nail bed bleedings of unknown etiology causing swelling and pain. At the age of 21 years, he was also diagnosed with hypogonadotropic hypogonadism with the following serum hormone concentrations: testosterone, 7.9 mmol/L (reference, 7.2–24 mmol/L); follicle‐stimulating hormone, 18.4 IU/L (reference, 0.7–11.1 IU/L); luteinizing hormone, 12.5 IU/L (reference, 0.8–7.6 IU/L). On ultrasound examination, testicular volume was estimated to 3–4 ml for each, corresponding to pre‐pubertal size.

**Table 1:** Overview of the clinical presentation of affected individuals FA.II‐1, FA.II‐2, and FB.II‐2

Features	FA.II‐1	FA.II‐2	FB.II‐2
Country of origin	Pakistan	Pakistan	Germany
Gender	Male	Female	Female
Age at last evaluation (years)	21	18	11
Birth measurements	2040 g; OFC: 33 cm	2985 g; L: 50 cm; OFC: 34 cm	NR
Neurological			
Developmental delay/hypotonia	Yes	Yes	Yes
Oculomotor apraxia	Yes	Yes	Yes
Cerebellar ataxia/wide‐based gait	Yes	Yes	Yes
Central breathing pattern abnormality during infancy	Yes	Yes	Yes
Intellectual disability	IQ: 64	IQ: 63	Yes
Skeletal			
Postaxial hexadactyly	No	Left hand	All limbs
5th	Yes	Yes	Yes
Otorhinolaryngo and respiratory			
Excessive expectoration	Yes	Yes	Yes
Dyspnea	Yes	Yes	NR
Chronic sinusitis and rhinitis	Yes	Yes	Yes
Chronic otitis media	Yes	No	Yes
Ectodermal			
Dental anomalies	Yes	No	No
Skin hypopigmentation	No	No	Yes

Abbreviation: NR, not reported; OFC, occipitofrontal head circumference.

FA.II‐2 was born at term with left hand postaxial hexadactyly and fifth finger clinodactyly and normal birth measurements. Episodes of tachypnea were noted from infancy to early childhood and muscular hypotonia was present at an early age. She was referred for ophthalmological examination due to poor eye contact and peculiar thrusting of the head to one side with the eyes deviating to the opposite side, and was later diagnosed with saccadic initiation failure, also known as oculomotor apraxia. Cerebral MRI examination at 7 months, repeated at 6 and 18 years, revealed similar cerebellar abnormalities as in FA.II‐1, compatible with JBTS (Figure 1c). Delayed psychomotor development became evident from an early age, but with extra support she was able to attend regular school. Assessment with Wechsler Intelligence Scale for Children (WISC) IV at the age of 16 years showed FSIQ IQ 63 (confidence interval, 59–69) compatible with mild intellectual disability. Anthropometric measurements have been within normal range. Similar to her brother, she suffered from nasal congestion and frequent upper respiratory tract infections accompanied by dyspnea, wheezing, and cough. Chronic sinusitis with retention of mucoid material (Figure S1) necessitated endoscopic sinus surgery. At 14 years, the concentration of nasal nNO was 616 ppb (normal). However, chest computed tomography (CT) indicated bronchial wall thickening and her sputum culture became positive for *Haemophilus influenzae* and *Pseudomonas aeruginosa*. Later, dyspnea and reduced working capacity became increasingly evident and lung function tests were pathological. She also suffered from a number of abnormalities and diseases without obvious connection to the JBTS, for example, nasal septum deviation, which was corrected by surgery (Table S1). Over the last 8 years, she needed more than 30 ophthalmological visits due to recurrent keratoconjunctivitis, a condition of uncertain etiology, which was treated with topical steroid ointment and artificial tears. Notably, keratoconjunctivitis (or blepharokeratoconjunctivitis) is reported to be more frequent in the Indian–Pakistani population (Viswalingam et al., 2005). She had urinary incontinence and recurrent urinary tract infections including pyelonephritis. No osmotic disturbance was detected. Ultrasound and CT examinations of the renal tract were normal, including kidney size. From the age of 16 years, she complained of debilitating headaches.

FA.II‐3, a female patient, was noted from an early age to have microcephaly and developmental delay. She also had bilateral cataracts (Table S1). Due to JBTS in the two elder siblings her development was followed closely. However, she was not noted to exhibit signs of oculomotor apraxia or ataxia and cerebral MRI was normal (Figure 1d). At age 12 years, she was assessed with WISC‐V showing full scale IQ 69. At 13.5 years, lack of secondary sexual characteristics lead to pelvic MRI and ultrasound examinations disclosing the absence of uterus and ovaries, and endocrinological assessment indicated primary ovarian insufficiency. She had normal feminine karyotype analysis and whole‐exome sequencing (WES) did not reveal relevant abnormalities. In view of neuromuscular features in FA.II‐1, CK was measured on several occasions, but was concluded to be normal (Table S1).

FA.II‐4, a female, had microcephaly, bilateral cataract (operated) and nasal septum deviation (awaiting operation). At the age of 9 years, she started to complain of muscular pain and weakness. CK concentrations were found to be elevated when measured on four occasions ranging from 305 to 460 U/L (reference < 200 U/L). The neurophysiological examination revealed motor and sensory neuropathy. MRI examination of the pelvis and lower extremities showed deposition of fat in the musculature in a similar fashion as in FA.II‐1. However, muscle biopsy did not show myopathy and fat infiltration but was still interpreted as pathological with mainly neurogenic changes. At the age of 10 years, pelvic MRI examination revealed agenesis of the ovaries and the uterus. An overview of the clinical features are available in Table S1.

The clinical picture of FA.II‐1 and FA.II‐4 were similar in regard to muscular pain and weakness, and CK elevation and the same neuromuscular disease. For comparison of features of hypogonadism, see Table S1.

In addition to her live children, mother FA.I‐2 had two first trimester miscarriages; prenatal ultrasound scanning and autopsy were not performed. The last pregnancy of FA.I‐2 concluded with an in utero death at 15 weeks (FA.II‐5 in Figure 1a). On ultrasound scan, this fetus presented with dolichocephaly, scaphocephaly, and a thick nuchal fold. Fetal DNA of FA.II‐5 tested negative for chromosomes 13, 15, 16, 18, 21, X, and Y aneuploidies by quantitative fluorescence polymerase chain reaction technique.

The parents in Family B (FB; Figure 1e) were first cousins of German origin. The mother reported a total of six first trimester miscarriages. Karyotyping was performed in one of these fetuses and yielded a normal male result (46,XY). Five years after the birth of a healthy daughter, the Family B proband was born; she was 10 years old at last clinical exam. She was born at 38 weeks of gestation by cesarean section and birth measurements were within the normal range. Postaxial hexadactyly was present on all four extremities (Figure S1). Hypopigmented areas were irregularly scattered over her entire body (Figure S1). Abnormal eye movements and psychomotor delay were noted from an early age. The muscle tone was reduced, and consequently, she wore splints during the day. Both eyes showed normal findings on funduscopy without evidence of retinal dystrophy and electroretinography was normal. Orthoptic examination revealed symptoms of Cogan II syndrome/oculomotor apraxia (MIM #257550). Horizontal saccades were abnormal, while vertical saccades showed no deficits. She used head thrusts to compensate for the inability to accomplish voluntary horizontal saccades. Oculomotor apraxia became gradually less severe during childhood. Diadochokinesis was shown to be delayed, indicating cerebellar dysfunction. Axial cerebral MRI examination at 5 years showed horizontally oriented superior cerebellar peduncles, vermis hypoplasia, and superior cerebellar foliar dysplasia (Figure 1f). Upper respiratory problems were present from an early age. She had a constant running nose and recurrent otitis media, requiring bilateral tympanostomy tubes at 7 years. At 8 years, nNO concentrations were found in the normal range. At age 11.5 years, height was 154.5 cm (75–90th percentile) and weight 58.6 kg (97th percentile), and OFC was 55 cm (90th percentile). She had learning difficulties and attends a school for children with special needs. The clinical diagnosis of FB.II‐2 was JBTS.

### 2.3 Experimental work

#### 2.3.1 WES and downstream data analysis

In Family A, genomic DNA from individuals FA.I‐1, FA.I‐2, FA.II‐1, FA.II‐2, FA.II‐3, FA.II‐4 was extracted from peripheral blood with the QIAsymphony SP (Qiagen). In FA.I‐2, FA.II‐1, FA.II‐2 exonic regions were enriched using SureSelectXT Human All Exon v5 (Agilent Technologies) with 3‐µg DNA input, as recommended by the manufacturer (protocol v.1.6). The samples were sequenced on an Illumina HiSeq2000 instrument (Illumina Inc.) with 100‐bp paired‐end reads. Alignment against the GRCh37 human reference genome was performed with a Burrows–Wheeler Aligner (BWA‐MEM, v.0.5.9; H. Li & Durbin, 2009). PCR duplicates were marked with Picard (v.1.104; http://broadinstitute.github.io/picard/); and indel realignment, base quality recalibration, and joint variant calling (UnifiedGenotyper) was performed with the Genome Analysis Toolkit (v.2.5; GATK; DePristo et al., 2011; McKenna et al., 2010). Functional annotation was performed with snpEff v.2.0.5 and Variant Effect Predictor (VEP) using Ensembl 71 (Cingolani et al., 2012; McLaren et al., 2016).

DNA from FA.I‐1 and FA.II‐3 was analyzed in a similar fashion, but exon enrichment was performed with SureSelectXT Human All Exon v6 with 1‐µg DNA input. Alignment was performed with BWA v.0.7.15 (H. Li & Durbin, 2009). PCR duplicates were marked with Picard (v.1.124; http://broadinstitute.github.io/picard/); and indel realignment, base quality recalibration, and joint variant calling (HaplotypeCaller) were performed with the Genome Analysis Toolkit (v.3.6; DePristo et al., 2011; McKenna et al., 2010). Functional annotation was performed with VEP using Ensembl 85 (Cingolani et al., 2012; McLaren et al., 2016).

The variant call files generated were analyzed using the Filtus program (Vigeland et al., 2016). We discarded variants with allelic frequency >0.01 in any of the databases used (gnomAD, ExAC, 1000Genomes, in‐house database of >250 whole exomes). We focused on single‐nucleotide variants (SNVs) and insertion–deletion variants (indels) predicted to be missense, nonsense, frameshift, or splicing variants. We discarded variants with a predicted pathogenicity score <15 according to the Combined Annotation‐Dependent Depletion (CADD) score (scaled C‐score; Kircher et al., 2014). Missense variants were also evaluated with the in silico tools of pathogenicity prediction PolyPhen2 (Adzhubei et al., 2013) and Sorting Intolerant from Tolerant (Ng & Henikoff, 2003).

In Family B, we utilized our customized targeted sequence capture approach previously described (Hoff et al., 2013; Lu et al., 2017). In short, a customized sequence capture library (NimbleGen) was designed that targeted exons and additional 35 bp of flanking intronic sequence, respectively. DNA was enriched using the NimbleGen SeqCap EZ choice sequence capture approach, and sequenced using Illumina HiSeq sequencing‐by‐synthesis technology with an average coverage of about 500×. For interpretation of generated NGS data, we have developed our own bioinformatic algorithms using a stepwise filtering process. Very recently, we added WES to exclude any other genetic finding that might add to the phenotype in this patient. However, we did not identify any other variant thought to be of pathogenic relevance.

NGS data generated in the above families were validated by Sanger sequencing. Primers were designed using Primer3Plus (Untergasser et al., 2012). PCR products were purified and Sanger‐sequenced using an ABI 3730xl DNA Analyzer and ABI BigDye Terminator Cycle‐Sequencing Kits v3.1 (Thermo Fisher Scientific). Sequences were analyzed with Applied Biosystems DNA Sequencing Analysis Software version 5.1 (Applied Biosystem) and SeqScape Software version 2.7 (Thermo Fisher Scientific).

In Family A, genomic DNA from FA.I‐1, FA.I‐2, FA.II‐1, FA.II‐2, FA.II‐3, and FA.II‐5 were tested using the following primer pair: Fwd, 5ʹ‐CACATAGCATCTCGCCCACAGTT‐3ʹ and Rev, 5ʹ‐GTCGGTCCACCTGCCTCTACAC‐3ʹ.

In Family B, FB.I‐1, FB.I‐2, and FB.II‐2 were tested using the following primer pair: Fwd, 5ʹ‐CATGAGTTTTGGTGGGGACATT‐3ʹ and Rev, 5ʹ‐GCAATGATATGGACGGAAAGG‐3ʹ.

#### 2.3.2 Studies on zebrafish as a vertebrate model system

##### 2.3.2.1 Zebrafish strains and maintenance

Zebrafish (*Danio rerio*) were raised under standard conditions at 28°C and zebrafish embryos were staged according to Kimmel (Kimmel et al., 1995). The following strains were used: AB/TL wild‐type, *Tg(wt1b:EGFP)* (Perner et al., 2007).

##### 2.3.2.2 Zebrafish microinjections

For synthesis of sense RNA, we cloned full‐length zebrafish cby1 into pCS2+. Sense RNA was prepared from ApaI‐linearized cby1‐pCS2+ using the SP6 mMessage mMachine Transcription Kit (Thermo Fisher Scientific). Morpholinos (MOs) and sense RNA were diluted in 0.1‐M KCl to concentrations of 4 and 0.02 μg/μl, respectively. One nanoliter of this dilution was injected through the chorion of 1‐cell stage embryos. To attenuate possible off target effects, a p53 MO (Robu et al., 2007) was coinjected 1.5‐fold to the other MOs used. The following translation/splicing‐blocking (TB/SB) antisense MOs (Gene Tools; www.gene-tools.com/morpholino; antisense oligos) were used: TB‐MO *cby1* (GTCTGACTTCTTAACCAAACGTGGA), SB‐MO *cby1* (ACAGAAACGTGTACTTACTGTATGT), and a standard control (Co)‐MO.

We used the online tool (http://crispr.mit.edu/) to design efficient guide RNAs (gRNA) targeting genomic *cby1* in zebrafish. We used a gRNA targeting the sequence 5ʹ‐AGCGATGCAGACTTTCGAGGTGG‐3ʹ (protospacer adjacent motif sequence underlined) in exon2 of *cby1*. We cloned double‐stranded oligos (*cby1_gRNA_F*: 5ʹ‐TAGGAGCGATGCAGACTTTCGAGG‐3ʹ and *cby1_gRNA_R*: 5ʹ‐AAACCCTCGAAAGTCTGCATCGCT‐3ʹ) into BsmBI linearized pT7‐gRNA (Addgene). *cby1‐gRNA* mRNA was synthesized from BamHI‐HF linearized pT7‐cby1‐gRNA using the MEGAshortscript T7 Transcription Kit (Ambion). One nanoliter containing Cas9 protein (300 ng/µl; PNA‐BIo) and *cby1‐gRNA* mRNA (100 ng/µl) was injected into 1‐cell stage *Tg(wt1b:EGFP)* embryos that were raised to adulthood. To confirm successful indel formation, the genomic DNA of 1 day post fertilization (dpf) embryos was amplified using the primers *cby1_gRNA_ID_F* (5ʹ‐ATGTTGTCGATTTACGCTTGTG‐3ʹ) and *cby1_gRNA_ID_R* (5ʹ‐GCGGTTAAAACATGGTCAATTT‐3ʹ). Sanger‐sequenced PCR products were analyzed using the inference of CRISPR (clustered regularly interspaced short palindromic repeats) edits from Sanger trace data (ICE) method (Hsiau et al., 2019). Potential founders were outcrossed to *Tg(wt1b:EGFP)* and the offspring was analyzed for mutations in *cby1*. Embryos from identified founders were raised to adulthood, followed by outcrosses and the identification of *cby1* mutations in the offspring. This resulted in the identification of several different mutations in *cby1*. For our experiments, we have chosen an indel mutation that results in the deletion of one nucleotide and the insertion of two nucleotides resulting in a frame‐shift and premature stop codon of Cby1. Maternal‐zygotic (MZ) *cby1* mutants (MZ*cby1*) were obtained from incrosses of homozygous carriers.

##### 2.3.2.3 Reverse‐transcription polymerase chain reaction (RT‐PCR) analysis

Semiquantitative RT‐PCR was performed to determine expression of zebrafish *cby1* during embryonic development and in adult organs. Total RNA from entire zebrafish embryos or adult zebrafish organs was extracted with the RNeasy Kit (Qiagen), followed by complementary DNA (cDNA) synthesis with the ProtoScript First Strand cDNA Synthesis Kit (Promega). Analysis of zebrafish *ef1α* was used as a loading control. The following primers were used for PCR analysis: *cby1* (forward: 5ʹ‐GCTAACGGTAGTCTCACCTCCA‐3ʹ; reverse: 5ʹ‐ CACCAATGTTCATCACAGGAGA‐3ʹ), *ef1α* (forward: 5ʹ‐ATCTACAAATGCGGTGGAAT‐3ʹ; reverse: 5ʹ‐ATACCAGCCTCAAACTCACC‐3ʹ).

##### 2.3.2.4 Quantitative real‐time PCR (qPCR)

Total RNA was obtained from 30 homozygous MZ*cby1* mutant zebrafish embryos or respective control at 1 dpf using the RNeasy Kit. First strand cDNA synthesis was performed using the ProtoScript First Strand cDNA Synthesis Kit. qPCR was performed on a Light Cycler 480 (Roche) using the DyNAmo Flash Sybr Green Kit (Thermo Fisher Scientific). *ef1α* was used as normalization control. Technical triplicates of four biological samples were analyzed for gene expression. The following primers were used for qPCR analysis: *ef1α* (forward: 5ʹ‐TGCCAACTTCAACGCTCAGGTC‐3ʹ; reverse: 5ʹ‐TCAGCAAACTTGCAGGCGATG‐3ʹ), *gli1* (forward: 5ʹ‐TCAGACGTCCTCTCGCCTTA‐3ʹ; reverse: 5ʹ‐AGCTCATGTCTCCGATTGCC‐3ʹ), *ptc1* (forward: 5ʹ‐GGGTCCTGAATGGACTGGTG‐3ʹ; reverse: 5ʹ‐CCGCTGGAGATACCTCAGGA‐3ʹ), *axin2* (forward: 5ʹ‐ACCCTCGGACACTTCAAGGA‐3ʹ; reverse: 5ʹ‐GTGCAGTCATCCCAGACCTC‐3ʹ), *wnt8a* (forward: 5ʹ‐ATTCGTGGATGCGCTTGAGA‐3ʹ; reverse: 5ʹ‐TTACAGCCAAACGTCCAGCTT‐3ʹ).

##### 2.3.2.5 Whole mount in situ hybridization (ISH) analysis and immunostaining

ISH was performed as previously described (Lu et al., 2017). Digoxigenin‐labeled antisense and sense RNA were prepared from NotI‐linearized full‐length cby1‐pCS2+ using T3 RNA polymerase (Roche) and XhoI‐linearized full‐length cby1‐pCS2+ using SP6 RNA polymerase (Roche, Basel, Switzerland), respectively. Whole mount immunostaining was performed as previously described (Hoff et al., 2018).

##### 2.3.2.6 Immunoblotting

For each condition, 30 dechorionated zebrafish embryos at 1 dpf were deyolked by incubation in calcium‐free Ringer's solution (116‐mM NaCl, 2.9‐mM KCl, 5‐mM HEPES, pH 7.2) containing 2‐mM Na_3_VO_4_ and protease inhibitors (Roche) for 10 min on ice followed by pipetting 10 times up and down and three washing steps with Ringer's solution to remove the yolk. Deyolked zebrafish embryos were lysed in 2 × sodium dodecyl sulfate (SDS) protein sample buffer (0.125‐mM Tris‐HCl [pH 6.8], 4% SDS, 20% glycerol, 10% β‐mercaptoethanol, 0.004% Bromophenol Blue) and heated for 5 min at 95°C. Proteins were separated by sodium dodecyl sulfate‐polyacrylamide gel electrophoresis, transferred to polyvinylidene fluoride membrane, and incubated with anti‐CBY1 (MBS9206551, MyBiosource, 1:2000) or anti‐gamma‐Tubulin (clone GTU‐88, Sigma Aldrich, 1:5000) and respective HRP‐conjugated antibodies (DAKO, 1:5000) and finally with immunoblot detection reagent (Perbio Science, Thermo Fisher Scientific).

##### 2.3.2.7 Microscopy and image acquisition

Brightfield images of whole mount in situ embryo stains were taken using an Axioplan2 microscope with Axiocam camera and using Axiovision software (Carl Zeiss). Embryos of the *Tg(wt1b:EGFP)* line were analyzed under a Leica MZ16 stereomicroscope (Leica), and nonconfocal fluorescent images were taken with a SPOT Insight Fire Wire System (Diagnostic Instruments). All images were exported as TIFF files and imported into Adobe Photoshop software CS2 to arrange figures. Confocal images of whole mount zebrafish immunostainings were generated with a Carl Zeiss LSM510 laser scanning microscope. Confocal z‐stacks were projected to one plane (maximum intensity projection).

##### 2.3.2.8 Measurement of ciliary length

Ciliary length was determined automatically using ImageJ Fiji (https://fiji.sc/) after running the following commands in macromodus on the maximum intensity projections of the confocal stacks: setThreshold(71, 255); setOption(“BlackBackground”, true); run(“Convert to Mask”); run(“Skeletonize”); run(“Analyze Particles…,” “size = 3 − Infinity show = Masks display add”); roiManager(“Measure”); overlapping cilia that could not clearly be distinguished from each other were manually excluded for the quantification.

##### 2.3.2.9 Statistical analysis and quantification

All data represent results from one of at least three independent experiments, which showed consistent results. Numbers of embryos used for analysis are indicated in the respective bar chart. Data were analyzed by Student's *t* test (two‐sided, unpaired); error bars represent the standard error of the mean (*SEM*). Immunoblot signals were quantified using Gel‐Pro Analyzer 6.0, INTAS and normalized to respective loading controls. Statistical analysis for qPCR results was performed using the GraphPad Prism software and significance was calculated with the one‐sample *t* test.

#### 2.3.3 Studies on fibroblast cultures

##### 2.3.3.1 Cell culture

Skin fibroblasts obtained from individual FA.II‐1, FA.II‐2, and FB.II‐2 and from three controls were cultivated in DMEM (Life Technologies) supplemented with 10% fetal bovine serum (FBS) and 1% penicillin–streptomycin.

##### 2.3.3.2 Immunofluorescence (IF) analysis

Fibroblasts between passages 4 and 9 were seeded in six‐well plates (BD Bioscience), 60,000 cells in each well, allowing growth on sterilized glass cover slips (18:1014/10; Hecht‐Assistent). To induce cilia formation, cells were serum‐starved for 24 or 72 h with Dulbecco's modified Eagle's medium (DMEM) supplemented with 1% penicillin–streptomycin without FBS. Then the cells were fixed for 10 min in 4% formaldehyde and then in 100% methanol at −20°C or directly in 100% methanol at −20°C or 4% formaldehyde and 100% cold methanol depending on the protocol of the primary antibodies used. To stimulate Hedgehog signaling pathway, cells were treated with Smoothened (SMO) agonist (SAG). Cells were seeded at ~80% confluence in complete medium for 24 h, and serum‐starved for 72 h, during the last 24 h of serum starvation 100 ‐nM of SAG (item no. 11914; Cayman Chemicals) was added and left for 24 h. The same volume of the solvent dimethyl sulfoxide (1 µl/ml medium) was added to cells not receiving SAG. Serum‐starved fibroblasts with and without SAG stimulation were used in immunofluorescence experiments to study SMO translocation.

##### 2.3.3.3 Staining protocol

Cells fixed on cover slips were washed twice with 1× PBS and then blocked for 15 min in 1× PBS containing 1% FBS and 0.5% Triton X‐100 (PBS‐AT) in a humid chamber. All antibody incubations were performed in PBS‐AT. The primary and secondary antibodies are listed in Table S2. After blocking with PBS‐AT, cells were incubated with primary antibodies for 2 h at room temperature in a humid chamber, washed with 1× PBS, and incubated with fluorescence‐labeled secondary antibodies for 45 min. Then, the secondary antibody was washed off with 1× PBS and cells were counter stained for DNA with Hoechst 33258 (Sigma‐Aldrich), washed with 1× PBS and then washed with distilled water to remove the salts, air‐dried, and mounted on slides using ProLong Gold (Life Technologies).

##### 2.3.3.4 Microscopy, image acquisition, and statistical analysis of immunofluorescence

For confocal imaging all images were captured using appropriate optical filter settings on a multifluorescent submicron bead‐calibrated (Life Technologies) AxioImager Z1 ApoTome microscope system (Carl Zeiss) equipped with plan‐apochromat lenses (×100/NA1.40; ×63/NA1.40; ×40//NA0.95) and Zeiss AxioCam MRm camera. To display the entire cell volume, images were presented as maximal projections of z‐stacks. Acquisition and analysis were done using AxioVision 4.8.2_SP2 (Carl Zeiss) software. For quantitative immunofluorescence analyses images were captured using appropriate optical filter settings on a multifluorescent submicron bead‐calibrated CellObserver microscope system (Carl Zeiss) equipped with a ×40/NA1.3 plan‐apochromat lens and a Zeiss AxioCam MRm camera. Images were processed using Zen 2.3 Blue edition (Carl Zeiss). Downstream analysis of the immunofluorescence images and signal quantification was done. For the quantification of the signal intensities, a region of interest (ROI), which included the cilium, was selected. The actual signal intensity of the ROI was obtained by subtracting the signal intensity of the background (outside the cell) to the signal intensity of the ROI. All intensity measurements were performed using ImageJ Fiji version (Schindelin et al., 2012). Statistical analysis of the immunofluorescence data was performed using GraphPad Prism version 7.02 for Windows (GraphPad Software) or ggplot2 (https://ggplot2.tidyverse.org), as indicated. Data were analyzed by unpaired *t* test with Welch's correction. Error bars represent *SEM*.

##### 2.3.3.5 RNA sequencing

Skin‐derived fibroblast cells from the three patients and three controls were cultured in DMEM supplemented with 10% FBS, 1% penicillin–streptomycin. Cells between passages 4 and 9 were used in experiments. Serum starvation (72 h) of the fibroblasts was used to induce ciliogenesis. The cells were washed with 1× PBS on the plate before treatment with trypsin and centrifugation. RNA extraction from cells was performed using the Paris™ Kit (Life Technologies) following manufacturer's instructions. RNA concentration of each sample was measured on a Qubit fluorometer with RNA HS Assay Kit and integrity of the RNA was assessed on an Agilent 2100 Bioanalyzer using RNA 6000 Nano Chips (Agilent Technologies).

Samples were prepared for RNA sequencing with the Illumina TruSeq Stranded mRNA‐seq sample prep (Illumina), which employs poly‐T beads to enrich the polyadenylated fraction of mRNA. The resulting libraries were indexed, pooled, and sequenced on an Illumina NextSeq 500 (Illumina) run with 75‐bp single reads, for a total of 463,062,019 reads (3.6–5.9 × 10^6^ reads per sample). Reads were aligned to the Ensembl gene model GRCh37 (homo_sapiens_ensembl_hg19.gf; Flicek et al., 2014) with HISAT2 2.0.1 (Kim et al., 2015).

For the bioinformatic analysis of RNA sequencing data, a count‐based statistical method was performed in R, using DESeq2 version 1.20.0 (M. I. Love et al., 2014) from the Bioconductor project (http://bioconductor.org). The gene model used for counting reads were taken from the Ensembl GTF file (http://www.ensembl.org/info/data/ftp/) for the GRCh37 model. Using the bioconductor package GenomicAlignments (http://bioconductor.org/packages/GenomicAlignments), reads were counted across genes. Patient and control read counts across the *CBY1* gene were noted and graphically displayed.

## 3 RESULTS AND DISCUSSION

### 3.1 **Genetic studies: Identification of homozygous variants in** *CBY1*

We performed WES of the affected siblings, FA.II‐1 and FA.II‐2, and their healthy parents (and also of individual FA.II‐3; Table S3, panel A,B). Analysis of the data under a recessive model, and further Sanger sequencing of available family members, identified a variant in *CBY1* Chr22(GRCh37):g.39067079_39067080del; GenBank: NM_015373.3:c.189_190del; p.(Val65*), with a CADD score (scaled C‐score) of 34. This change was homozygous in FA.II‐1 and FA.II‐2; heterozygous in the parents, and in FA.II‐3, FA.II‐4, andFA.II‐5 (Figure 1a). The *CBY1* c.189_190del variant (rs747514855) is observed with an allele frequency of 3.579e − 5 according to the Genome Aggregation Database (gnomAD) and of 4.942e – 5 according to the Exome Aggregation Consortium (ExAC), but importantly, no homozygotes have been reported. This variant is present in ClinVar (https://www.ncbi.nlm.nih.gov/clinvar/9) with the following ID: SCV001431226.

Targeted next‐generation sequencing gene panel testing in FB.II‐2 did not identify any pathogenic variants in documented ciliopathy genes, but revealed a homozygous *CBY1* variant Chr22(GRCh37):g.39064123_39064124dup; GenBank: NM_015373.3:c.64_65dup; p.(Asn23Profs*24), CADD score (scaled C‐score) of 32, which was validated by Sanger sequencing and shown to be homozygous in the child and heterozygous in the parents (Figure 1e). This variant was not reported in ExAC or gnomAD. This variant is present in ClinVar with the following ID: SCV001433873. At a later stage, WES was performed in FB.II‐2; however, we did not identify any other variant thought to be of pathogenic relevance.

CBY1 (MIM #607757) localizes to the distal end of the mother centriole (Steere et al., 2012), where it has multiple functions. CBY1 facilitates recruitment of ciliary proteins, such as the axoneme protein ARL13B (MIM #608922) and the transition zone protein AHI1 (MIM #608894; Lee et al., 2014), both encoded by genes that cause JBTS when mutated (MIM #612291 and MIM #608629, respectively). CBY1 is required for the efficient recruitment of small vesicles and their subsequent fusion into ciliary vesicles, facilitating docking of the basal bodies to the plasma membrane (Burke et al., 2014; Voronina et al., 2009). CBY1 also antagonizes the transcriptional coactivator beta‐catenin, a key mediator of the canonical Wnt signaling pathway (Takemaru et al., 2003). *CBY1* was, therefore, considered a putative disease‐causing gene in the two families, who shared clinical features of a ciliopathy.

As the hypogonadism and the neuromuscular disease observed in Family A did not segregate with the CBY1 variant and cannot be easily ascribed to a ciliopathy, we reanalyzed the WES data available in search for additional disease‐causing genes. However, we did not identify additional relevant pathogenic variants for the neuromuscular disease (Table S3, panel C) and for the hypogonadism (Table S3, panel D).

Diagnostic microarray analyses in FA.II‐1 (180k aCGH + SNP; Agilent Technologies) did not reveal any potentially pathogenic copy number variation. We also reanalyzed WES or targeted gene sequencing data (as described in (Bachmann‐Gagescu et al., 2015)) of 638 families with various ciliopathy diagnoses of unknown genetic cause. We did not identify any additional families with biallelic variants in *CBY1* (Table S4), suggesting that CBY1 dysfunction is an ultrarare cause of a hitherto uncharacterized ciliopathy.

Interestingly, CBY1 p.Asn23Profs*24 was described in a study aiming at assessing the contribution of CBY1 to obesity (F. Q. Li et al., 2007). Obesity is documented in ciliopathies including JBTS (Thomas et al., 2015). CBY1 has been identified as a proadipogenic factor required for adipocyte differentiation and the p.Asn23Profs*24 was found in one control subject, which lead the authors to suggest that decreased CBY1 activity may have a protective effect for obesity (Van Camp et al., 2013). Patient FB.II‐2, homozygous for p.Asn23Profs*24, at age 11.5 years had weight of 58.6 kg, >97th percentile, and height of 154.5 cm, 75th percentile. Based on these growth measurements, FB.II‐2 was obese (https://www.cdc.gov/healthyweight/bmi/calculator.html). However, more individuals should be studied to draw a conclusion on the possible effect CBY1 p.Asn23Profs*24 on obesity.

### 3.2 Depletion of *cby1* in the zebrafish resulted in ciliopathy‐related phenotypes

**Figure 2:**
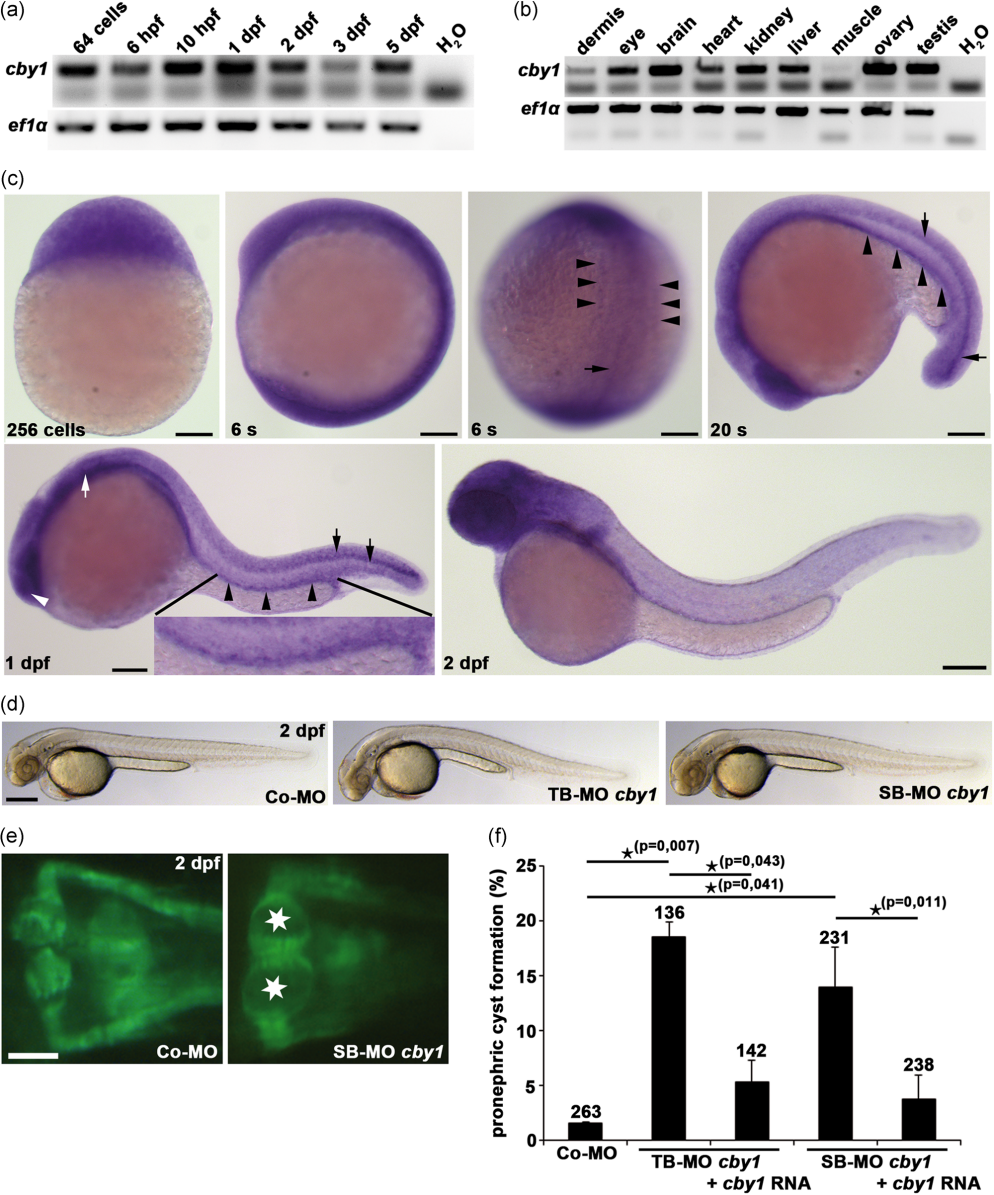
Zebrafish studies showing temporal and spatial expression of *cby1* and evidence of a ciliopathy phenotype after *cby1* knockdown. (a) Semiquantitative reverse‐transcription polymerase chain reaction (RT‐PCR) analysis reveals maternal expression of *cby1* at the 64‐cell stage and expression of *cby1* throughout embryogenesis. *ef1α* served as a loading control. (b) Semiquantitative RT‐PCR analysis reveals expression of *cby1* in most adult zebrafish organs analyzed, with prominent expression in eye, brain, kidney, liver, ovary, and testis. *ef1α* served as a loading control. (c) Whole mount in situ hybridization analysis for *cby1* in zebrafish. Maternal *cby1* transcripts are ubiquitously distributed at the 256‐cell stage. Broad expression of *cby1* at six‐somite stage (6s) with specific expression in axial mesoderm (black arrow) and pronephric mesoderm (black arrowheads). At 20‐somite stage (20s), *cby1* shows expression in the developing pronephric tubule (black arrowheads) and ventral spinal cord (black arrows). At 1 day post‐fertilization (1 dpf), expression of *cby1* is detected in ciliated organs including the nasal placode (white arrowhead), otic vesicle (white arrow), neural tube (black arrows), and pronephric tubule (black arrowheads). At 2 dpf, *cby1* has a broad expression in the head. Scale bars = 100 µm. (d) Brightfield images showing overall morphology of 2dpf zebrafish embryos injected with control morpholino (Co‐MO), translation‐blocking morpholino (TB‐MO) *cby1*, and splicing‐blocking morpholino (SB‐MO) *cby1*. Scale bar = 100 µm. (e) Knockdown of *cby1* results in pronephric cyst formation (stars), as shown in a dorsal view with anterior to the top of a *Tg(wt1b:EGFP)* zebrafish embryo at 2 dpf. Scale bar = 5 µm. (f) Quantification of pronephric cyst formation in 2dpf zebrafish embryos injected with Co‐MO, TB‐MO *cby1*, TB‐MO *cby1* + *cby1* mRNA, SB‐MO *cby1*, and SB‐MO *cby1* + *cby1* mRNA. There was significant prevention of pronephric cyst formation upon coinjection of *cby1* mRNA. The number of individual embryos analyzed is indicated above each bar

**Figure 3:**
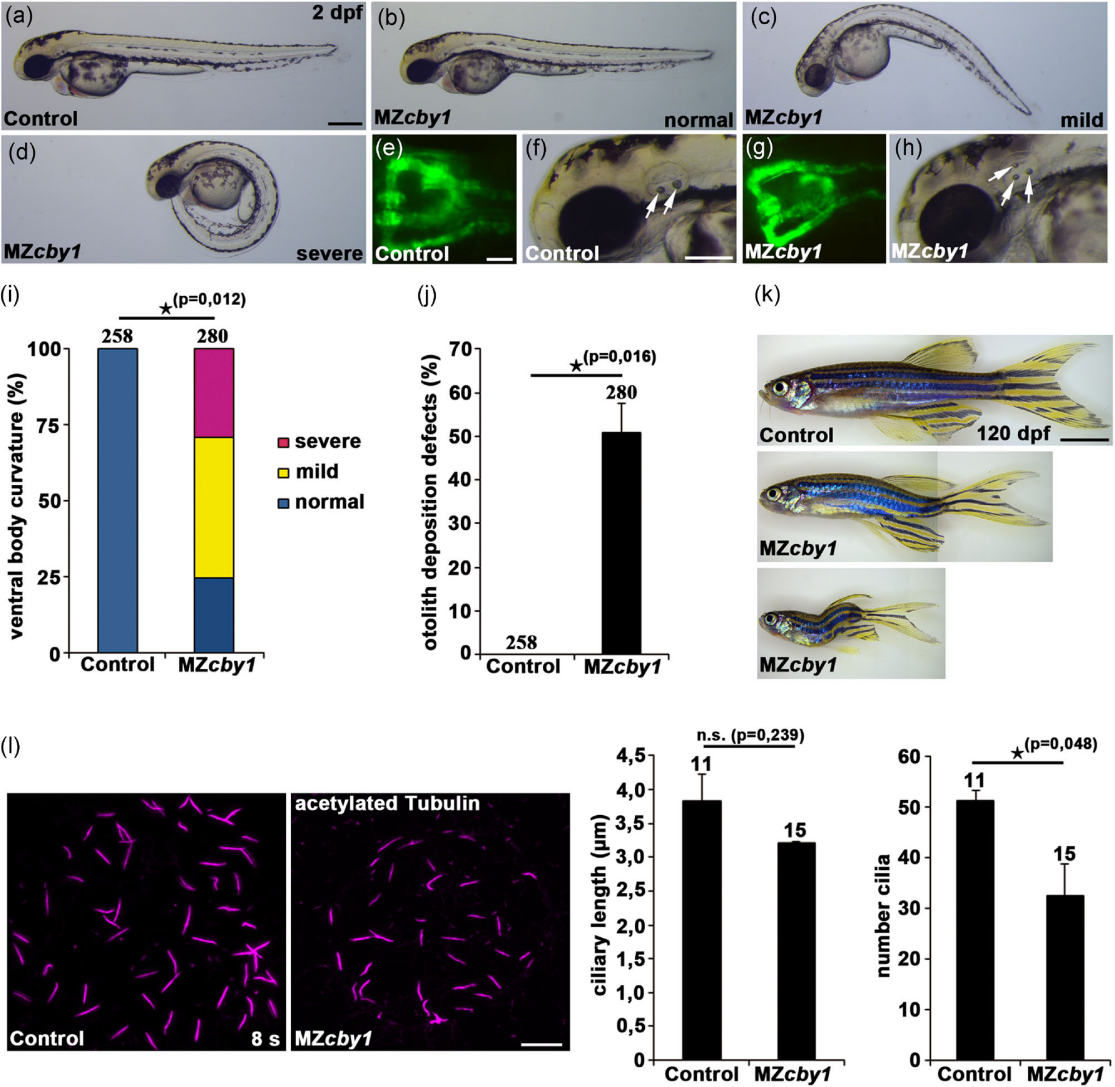
Zebrafish *cby1* knockout mutants display ciliopathy‐related phenotypes. (a–h) Images of maternal‐zygotic (MZ) *cby1* mutant embryos at 2 dpf display no or different degrees of ventral body curvature defects (b–d), no pronephric cyst formation as shown in a dorsal view with anterior to the top of a *Tg(wt1b:EGFP)* zebrafish embryo at 2 dpf (g), and prominent otolith deposition defects (white arrows) at 2 dpf (h) in comparison to the respective controls (a,e,f). Scale bars = 100 µm (a), 5 µm (e), and 50 µm (f). (i) Quantification of different degrees of ventral body curvature of 2dpf MZ*cby1* mutant embryos in comparison to the respective control. The number of individual embryos analyzed is indicated above each bar. (j) Quantification of otolith deposition defects of 2dpf MZ*cby1* mutant embryos in comparison to the respective control. The number of individual embryos analyzed is indicated above each bar. (k) Brightfield images of adult MZ*cby1* mutants at 120 dpf in comparison to the respective control. Scale bar = 500 µm. (l) Confocal images of the Kupffer's vesicle of control and MZ*cby1* mutant embryos at the stage of 8 somites (8s). Cilia were visualized by acetylated tubulin staining and ciliary length and number of cilia were quantified. Scale bar = 10 µm

We investigated the involvement of CBY1 in ciliary function using zebrafish, whose genome has only one *CBY1* orthologue. Zebrafish Cby1 protein displays a high degree of evolutionary conservation, with 71%–75% identity to its vertebrate counterparts (Figure S2). To determine the temporal and spatial expression pattern of *cby1* in zebrafish, we performed semiquantitative RT‐PCR and whole mount ISH analysis. The analysis revealed that *cby1* was expressed throughout zebrafish embryogenesis and in several organs of the adult zebrafish (Figures 2a–c and S2). We detected maternal *cby1* transcripts at early cleavage stages and revealed that *cby1* was expressed in several ciliated tissues including the nasal placode, otic vesicle, pronephric tubule, and spinal cord (Figure 2c). We then performed morpholino (MO) injections in zebrafish to study Cby1 in vivo functions (Figures 2d–f and S2). Knockdown of *cby1* with two validated MOs led to a ciliopathy‐related phenotype in zebrafish, as we detected pronephric cyst formation (Figure 2d–f). Coinjection of *D. rerio cby1* mRNA partially rescued pronephric cyst formation, thus confirming MO specificity (Figure 2f). Knockdown of *cby1* did not cause altered heart looping which is another well known ciliopathy‐associated phenotype (Figure S2). In addition to the *cby1* knockdown studies, we analyzed a CRISPR/Cas9‐induced *cby1* knockout in zebrafish (Figures 3, S2, and S3). MZ*cby1* mutant embryos displayed ciliopathy‐related phenotypes including ventral body curvature and otolith deposition defects (Figures 3a–j and S3). Other ciliopathy‐related phenotypes such as pronephric cyst formation or altered heart looping were not observed in the MZ*cby1* mutant embryos (Figures 3 and S2). A small proportion of the MZ*cby1* mutants is viable but displays phenotypic defects including smaller body size, shortened body axis, and different degrees of scoliosis at 120 dpf (Figure 3k). Acetylated tubulin immunostainings revealed defective ciliogenesis in MZ*cby1* mutant embryos including cilia in the Kupffer's vesicle, pronephric tubule, neural tube, and otic vesicle (Figures 3 and S3). It was previously shown that CBY1 antagonizes beta‐catenin and thereby affects the canonical WNT signaling pathway (D. Love et al., 2010; Steere et al., 2012; Takemaru et al., 2003; Voronina et al., 2009). In zebrafish MZ*oval* (*ovl*; *ift88*) mutants that lack all cilia, defects in Hedgehog, but not in Wnt signaling were observed (Huang & Schier, 2009). Through qPCR analyses, we, therefore, studied potential effects of zebrafish Cby1 loss of function on the expression of Hedgehog and Wnt signaling‐associated genes. In MZ*cby1* mutant embryos, we observed a significant reduction in *gli1* expression compared with respective control, *ptc1* was, however, not affected (Figure S3). Expression of the Wnt signaling genes *axin2* and *wnt8a* was not affected in MZ*cby1* mutant embryos (Figure S3). These results indicate that the ciliary defects in MZ*cby1* mutants partially impair Hedgehog signaling. The results in zebrafish support that reduced Cby1 expression causes a kidney defect, leading to a ciliopathy phenotype observed also in *Cby1* ^−/−^ mice (Lee et al., 2014). None of the three affected individuals described displayed gross renal anomalies. However, to further explore if and how loss of CBY1 affects the kidneys in humans, additional affected individuals are required for long‐term follow‐up.

### 3.3 Primary cilia of the affected individuals presented structural anomalies

**Figure 4:**
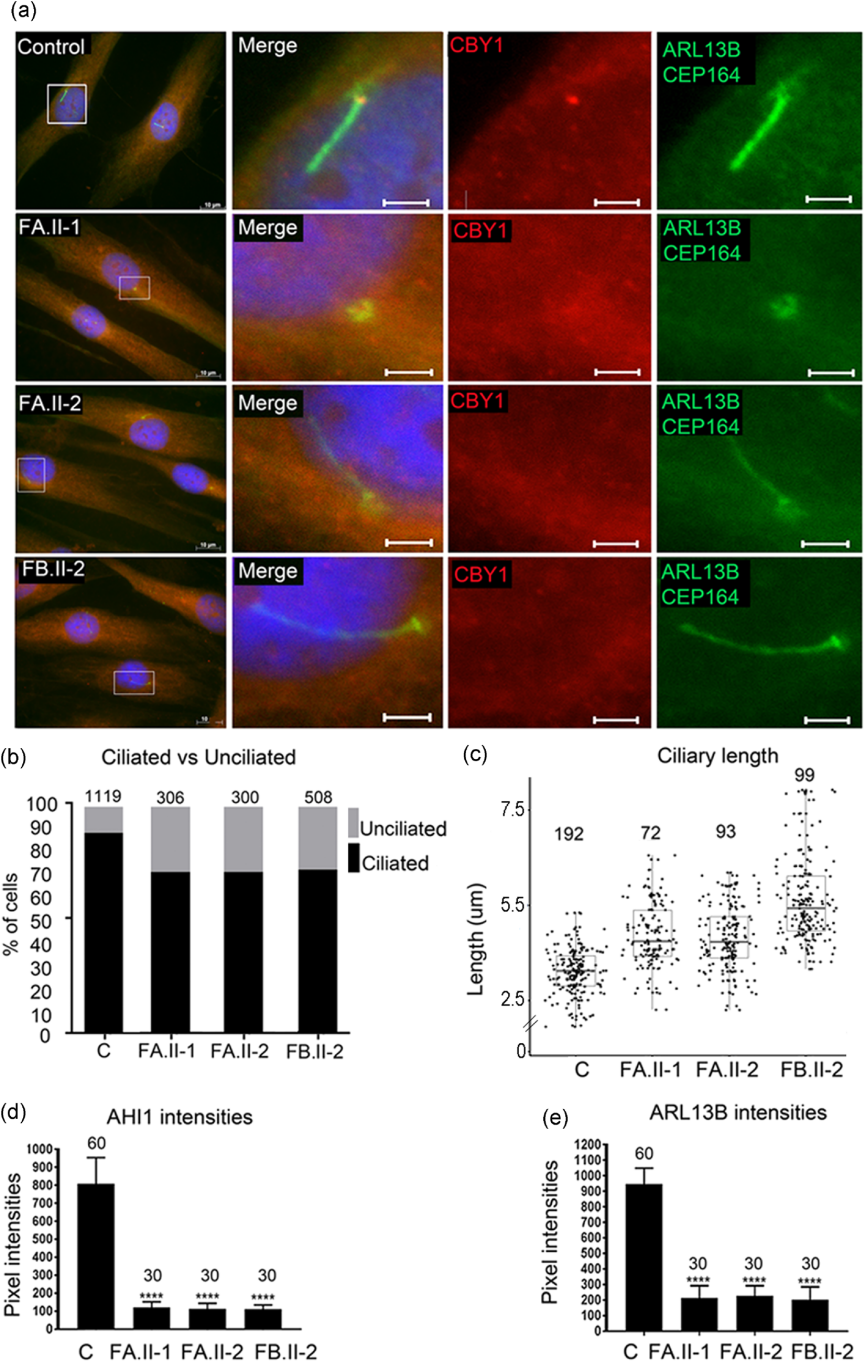
Reduced fraction of ciliated cells and increased primary cilia length in fibroblasts from patients. (a) Fibroblasts from two controls (images from one control shown) and from the individuals FA.II‐1, FA.II‐2, FB.II‐2 were fixed after 72 h of serum starvation and stained for ARL13B and CEP164 (Alexa488, green), CBY1 (Cy3, red), and nuclei (Hoechst, blue). The CBY1 signal was detected at the transition zone in the control, but not in cells from FA.II‐1, FA.II‐2, FB.II‐2. Scale bars are 10 µm in the left panel and 2 µm in the magnified pictures. (b) Fibroblasts from two controls and from the affected individuals FA.II‐1, FA.II‐2, FB.II‐2 were fixed after 72h serum starvation and stained for acetylated tubulin, CEP164, and nuclei (Hoechst). The numbers of ciliated fibroblasts were significantly reduced in FA.II‐1, FA.II‐2, and FB.II‐2 (*p* < .0001) compared with the two pooled controls (C in the figure). The numbers of cells analyzed are indicated on top of the bars. (c) Staining for ARL13B and polyglutamylated tubulin and nuclei (Hoechst) detected a significant increase in ciliary length in serum‐starved fibroblasts from the affected individuals FA.II‐1, FA.II‐2, FB.II‐2 compared with the two pooled controls (C). Median length difference between cilia in cells from affected individuals and control cells were 0.44, 0.43, and 1.65 µm, respectively (*p* < .0001). The numbers of cells analyzed are indicated on top. (d) Intensity measurement for AHI1 costained with polyglutamylated tubulin and Hoechst detected significantly reduced AHI1 signal intensities in serum‐starved fibroblasts from FA.II‐1, FA.II‐2, and FB.II‐2 compared with the two pooled controls (C) (*p* < .0001). The numbers of cilia analyzed are indicated on top of the bars. (e) Intensity measurements for ARL13B costained with polyglutamylated tubulin and Hoechst detected significantly reduced ARL13B signal intensities in serum‐starved fibroblasts from FA.II‐1, FA.II‐2, and FB.II‐2 compared with the two pooled controls (C) (*p* < .0001). The numbers of cilia analyzed are indicated on top of the bars

The *CBY1* frameshift variants identified in Families A and B may result in a mutated allele that undergoes nonsense‐mediated mRNA decay (NMD) or that encodes for a truncated protein. Quantification of the *CBY1* transcript in the RNA sequencing data revealed decreased levels in the patients compared with the controls, suggesting degradation of the mutated transcript through the NMD (Figure S4). To explore the cellular consequences of the *CBY1* variants, IF microscopy studies were performed in 72h serum‐starved fibroblast cultures established from FA.II‐1, FA.II‐2, and FB.II‐2, and from three controls. IF on ciliated fibroblasts using an antibody targeting the CBY1 amino acid residues 1–63, costained with the basal body marker CEP164 and the axoneme marker ARL13B, detected the CBY1 signal only in fibroblasts from controls (Figure 4a). The absence of any CBY1 signal in FA.II‐1, FA.II‐2, and FB.II‐2 fibroblasts suggested that the *CBY1* variants identified in Families A and B resulted in loss of CBY1 protein.

Reduced numbers of primary cilia were documented in embryonic fibroblasts from a *Cby1* ^−/−^ mouse model, accompanied by reduced levels of ARL13B and AHI1 (Lee et al., 2014). Therefore, we assessed by IF the fraction of ciliated cells and the ciliary structure in fibroblasts from FA.II‐1, FA.II‐2, and FB.II‐2 and from three controls using acetylated tubulin and CEP164 antibodies as ciliary markers. We detected a reduction in the number of ciliated fibroblasts (*p* < .0001) and significantly increased ciliary length (*p* < .0001) after serum starved for 72 h (Figure 4b,c) and for 24 h (Figure S5). IF also showed significantly reduced AHI1 (*p* < .0001) and ARL13B (*p* < .0001) signal intensities in cilia in the fibroblasts from FA.II‐1, FA.II‐2, and FB.II‐2 compared with controls after 72 h of serum starvation (Figures 4d,e, S6, and S7).

### 3.4 SMO recruitment was not affected in fibroblasts from the patients

One of the key functions of primary cilia is to coordinate signal transduction pathways including the Hedgehog signaling. This pathway plays a central role in early embryonic development by regulating cell growth, survival, differentiation, migration, and polarity. Sonic Hedgehog signaling is, for example, required for proper cerebellar patterning and maturation from early phases (De Luca et al., 2016); for early stages of lung development, including lung specification, primary bud formation, and branching morphogenesis (Fernandes‐Silva et al., 2017); and limb formation (Tickle & Towers, 2017). Dysfunction of many ciliary genes, including some JBTS‐causing genes, results in impaired Hedgehog signaling, and aberrant Hedgehog signaling has been proposed to underlie JBTS (Aguilar et al., 2012; Dafinger et al., 2011; Hynes et al., 2014; Shi et al., 2017). We, therefore, examined the effect of loss of CBY1 on SMO recruitment to the ciliary membrane in 72h serum‐starved fibroblasts before and after stimulation with the SMO agonist for 24 h. The fraction of SMO‐positive ciliated cells was 2%–4% without stimulation and 45%–51% upon SAG stimulation. No difference in the fraction of SMO‐positive cells was measured between cells from affected individuals compared with controls (Figure S8).

### 3.5 Studies of motile cilia did not reveal anomalies in the patients

Because all three affected individuals presented with airway symptoms, and *Cby1* ^−/−^ mice exhibited chronic upper respiratory infection and otitis media (D. Love et al., 2010; Voronina et al., 2009), we also analyzed motile cilia of the respiratory epithelium obtained by nasal brush biopsies in FA.II‐2 and FB.II‐2 by IF, transmission electron microscopy, and high‐speed video microscopy analysis. These experiments did not reveal any anomalies (Figure S9).

## 4 CONCLUSION

We describe biallelic *CBY1* LOF‐variants in three individuals presenting with clinical features of JBTS. In line with findings described for JBTS, we detected structural and functional cilia defects in fibroblasts from the affected individuals. These anomalies included increased length of primary cilia and a reduced fraction of ciliated fibroblasts. We also demonstrated that depletion of Cby1 in zebrafish causes ciliopathy‐related phenotypes. Comprehensive reanalysis of WES data obtained from a large cohort of individuals from different parts of the world with a clinical diagnosis of ciliopathy did not result in the identification of any additional putative biallelic pathogenic variants in *CBY1*, indicating that loss of CBY1 is most probably an ultrarare cause of disease.

## CONFLICT OF INTERESTS

Nicholas Katsanis is a paid consultant for and holds significant stock of Rescindo Therapeutics, Inc. Eva Decker, Nadine Bachmann, and Carsten Bergmann are employees of the Limbach group for which Carsten Bergmann heads and manages Limbach Genetics. In addition, Carsten Bergmann holds a part‐time faculty appointment at the University of Freiburg. The remaining authors declare that there are no conflict of interests.

## AUTHOR CONTRIBUTIONS

Daniel Epting, Lokuliyange D. S. Senaratne, Elisabeth Ott, Asbjørn Holmgren, Dulika Sumathipala, Julia Wallmeier, Diana Bracht, Kari‐Anne M. Frikstad, Tuva Barøy, Christian Decker, Soeren S. Lienkamp, Sebastian Patzke, Manuela Zucknick, Heymut Omran, Eirik Frengen, Carsten Bergmann, and Doriana Misceo contributed to the acquisition, analysis, or interpretation of the data. Daniel Epting, Lokuliyange D. S. Senaratne, Carsten Bergmann, and Doriana Misceo drafted the manuscript. Ian G. Phelps, Dan Doherty, Rachel Giles, Miriam Schmidts, Erica E. Davis, Nicholas Katsanis, Dan Doherty, Eva Decker, Nadine Bachmann, and Carsten Bergmann screened the cohorts of patients. Selma M. Larsen, Suzanne Crowley, Alma Sikiric, Barbara Käsmann‐Kellner, Eva Decker, Nadine Bachmann, Petter Strømme, and Carsten Bergmann recruited and investigated the patients. Petter Strømme evaluated the clinical and radiological and endocrinological and neuromuscular data/findings for Family A. Daniel Epting, Erica E. Davis, Dan Doherty, Petter Strømme, Eirik Frengen, Carsten Bergmann, and Doriana Misceo contributed to the critical revision of the manuscript for important intellectual content. Daniel Epting, Petter Strømme, Eirik Frengen, Carsten Bergmann, and Doriana Misceo conceived the study. All authors read and approved the final manuscript.

## Supporting information

Supporting information.

## Data Availability

Data available on request, but may be subjected to restrictions due to privacy/ethical restrictions.
