# Supplementary material for: Loss of CBY1 results in a ciliopathy characterized by features of Joubert syndrome
Source: Hum Mutat. 2020 Nov 1;41(12):2179–94. doi: 10.1002/humu.24127 (PMC7756669; doi:10.1002/humu.24127)
Supplement: Supplementary file 1 — Supporting information. [file HUMU-41-2179-s001.docx]

**Supporting information**

**
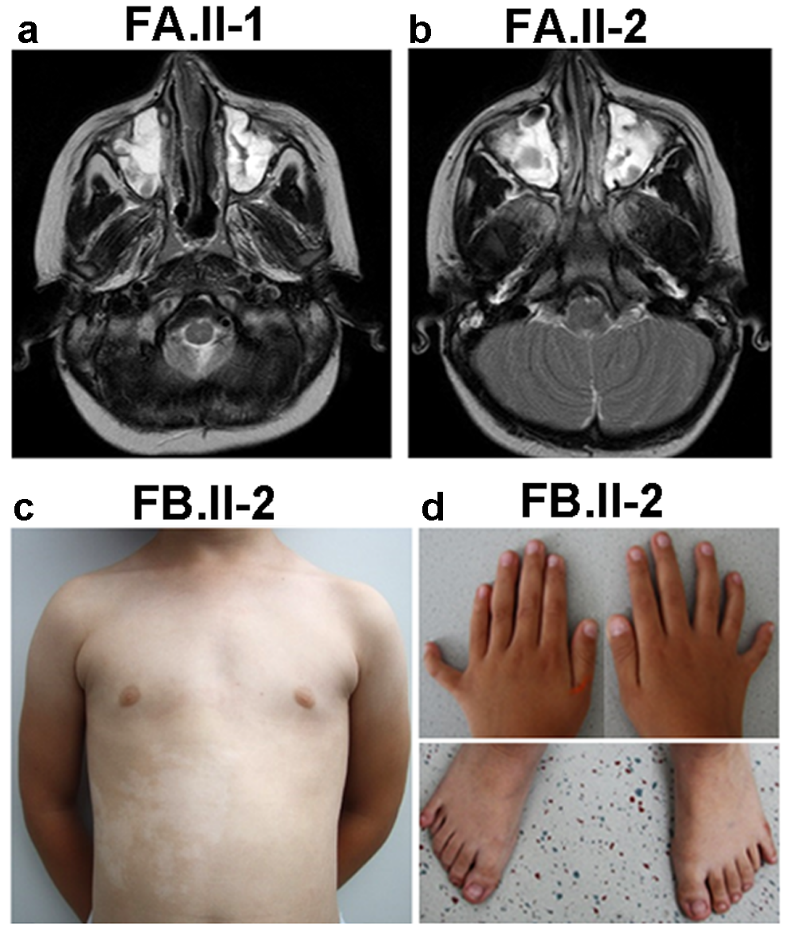
**

**Figure S1 Clinical details in the affected individuals from Family A and Family B.**

(a,b) T2 weighted axial view at the level of the brain stem and cerebellar hemispheres incidentally showing hyperintensity in the paranasal sinuses, filled with mucous in FA.II-1 and FA.II-2.

(c,d) Photograph of FB.II-2 at the age of 5 years. Note hypopigmented skin patches on the trunk, and the postaxial hexadactyly.

**
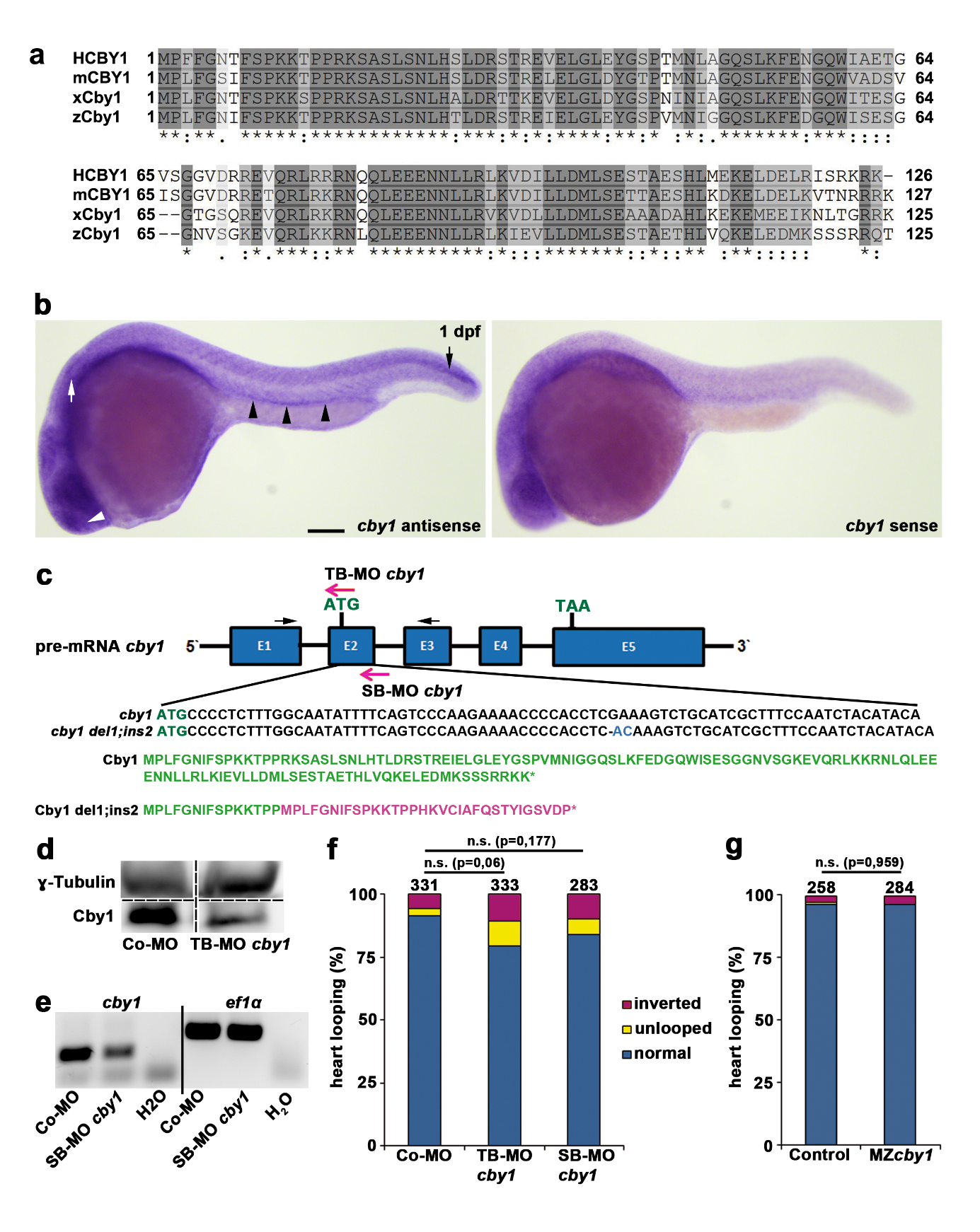
**

**Figure S2 Study of Cby1 in the zebrafish model.**

(a) Alignment of zebrafish Cby1 (zCby1) to orthologues from *Homo sapiens* (HCBY1), *Mus musculus* (mCBY1) and *Xenopus laevis* (xCby1) shows 71-75% amino acid conservation.

(b) Whole mount *in situ* hybridization analysis with either antisense or the respective sense RNA probe for *cby1* in zebrafish at 1 dpf. Specific *cby1* staining is only detectable in zebrafish embryos that were hybridized with the antisense RNA probe; nasal placode (white arrowhead), otic vesicle (white arrow), neural tube (black arrow) and pronephric tubule (black arrowheads).

(c) To knockdown zCby1 a translation blocking morpholino (TB-MO) was targeted at exon 2 at the translation initiation site and a splicing blocking morpholino (SB-MO) was targeted at intronic region between exon 2 and intron 2. CRISPR/Cas9-induced mutation of zebrafish *cby1*. Exon2 of zebrafish *cby1* is shown with the start codon ATG (green). The CRISPR/Cas9-induced *cby1* mutant displays an indel mutation (insertion of the two nucleotides AC (blue) and deletion of one nucleotide G) resulting in a frame-shift and premature stop codon in Cby1 mutants.

(d) Western blot indicates a reduction of Cby1 protein level to 12% when *cby1* was blocked with TB-MO compared to embryos injected with Control morpholino (Co-MO).

(e) Semiquantitative RT-PCR reveals significant reduction of the *cby1* level in embryos injected with SB-MO compared to Co-MO. *ef1α* served as a loading control.

(f) Quantification of heart looping in 2 dpf zebrafish embryos injected with Co-MO, TB-MO *cby1* and SB-MO *cby1*. The number of individual embryos analyzed is indicated above each bar.

(g) Quantification of heart looping in 2 dpf maternal zygotic (MZ) *cby1* embryos in comparison to the respective Control. The number of individual embryos analyzed is indicated above each bar.


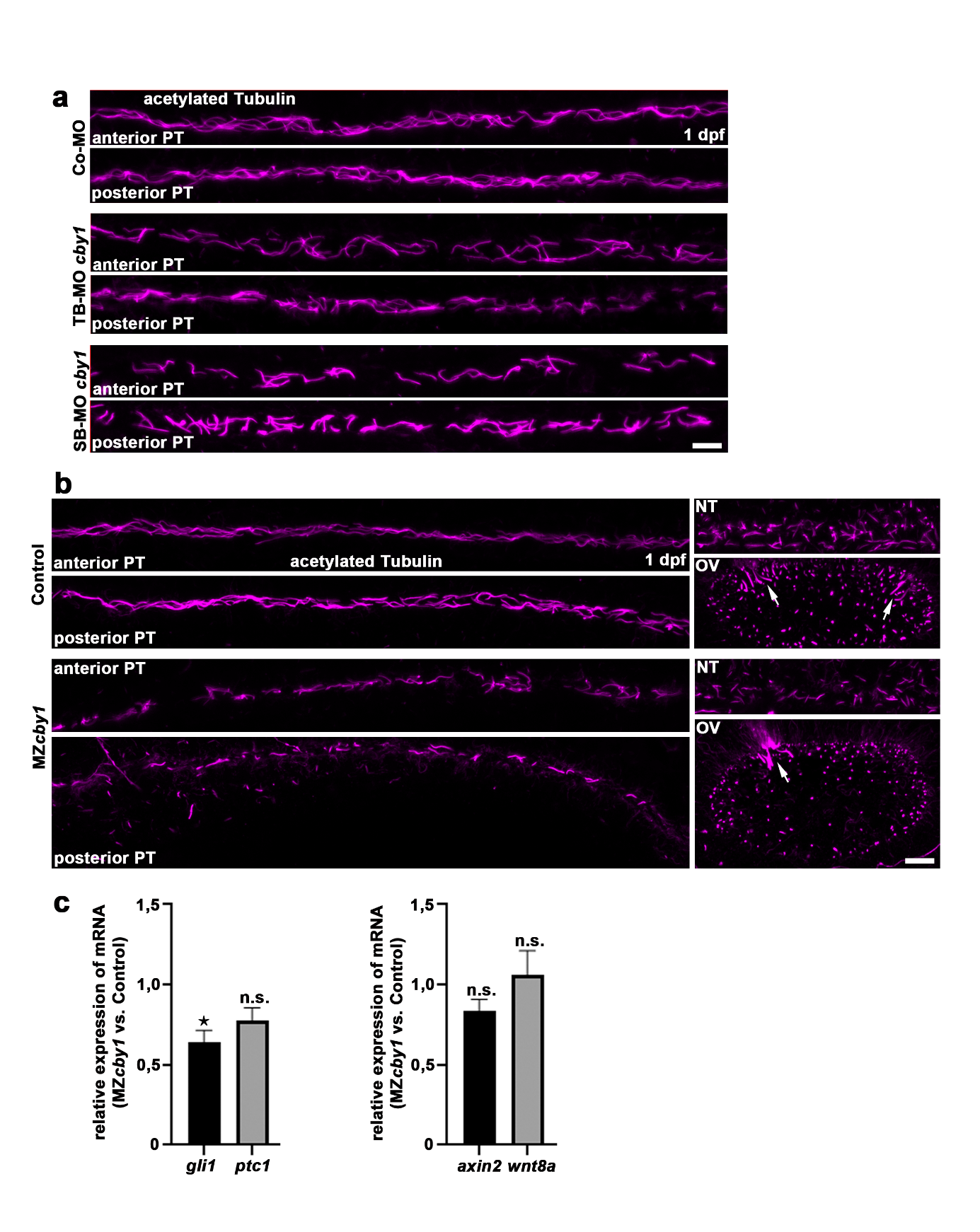


**Figure S3 Analysis of ciliogenesis and of Hedgehog and Wnt signaling in *cby1* depleted zebrafish embryos.**

(a) Acetylated Tubulin staining revealed defective cilia formation in the anterior and posterior part of the pronephric tubule (PT) of 1 dpf *cby1* morphant embryos in comparison to the Control.

(b) Acetylated Tubulin staining revealed defective cilia formation in the anterior and posterior part of the pronephric tubule (PT), the neural tube (NT) and otic vesicle (OV; white arrows mark longer tether kinocilia and motile cilia at the anterior and posterior poles of the otic lumen) of 1 dpf MZ*cby1* mutant embryos in comparison to the respective Controls.

(c) Quantification of the relative expression of Hedgehog (*gli1, ptc1*) and Wnt (*axin2, wnt8a*) signaling genes in MZ*cby1* mutant embryos compared to the respective Control at 1 dpf. MZ*cby1* mutants show statistically significant reduction in *gli1* expression (p=0,017). Expression of *ptc1* was not significantly (n.s.) affected (p=0,064). Wnt signaling was not significantly affected in MZ*cby1* mutants (*axin2*: p=0,107; *wnt8a*: p=0,719).


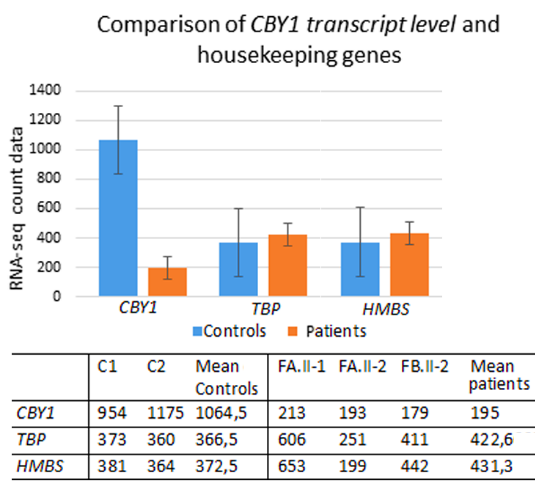


**Figure S4 RNA sequencing data from fibroblasts of patients and controls indicated reduced *CBY1* transcript levels in patient cells.**

All 14 housekeeping genes reported in https://www.genomics-online.com/resources/16/5049/housekeeping-genes/were analyzed in the RNA sequencing data. The read counts (representing gene expression level) in the RNA sequencing data from skin fibroblasts of the patients (FA.II-1, FA.II-2 and FB.II-2) were compared to the controls. *TBP* and *HMBS,* showed the closest count range to that of *CBY1*, and were therefore used for the comparison, which indicated that the *CBY1* transcript level was reduced in patient cells compared to controls.


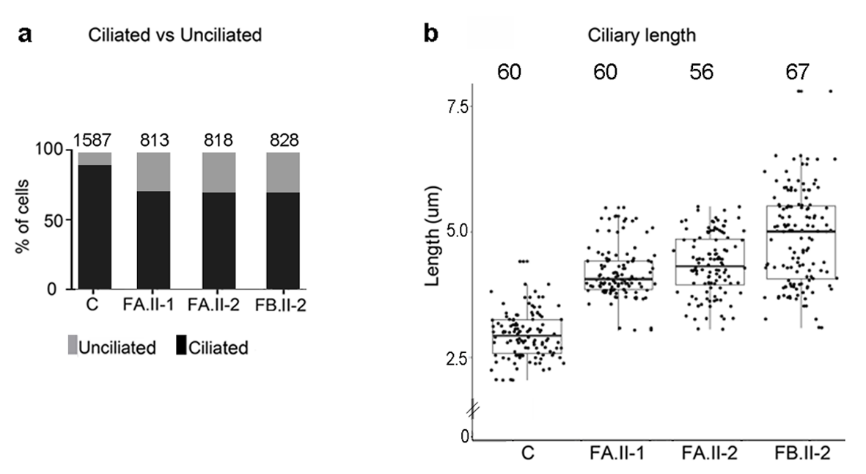


**Figure S5 Reduced fraction of ciliated cells and increased ciliary length in fibroblasts from patients compared to controls after 24 hours serum starvation.**

(a) Staining for acetylated Tubulin and CEP164 was used to count the numbers of ciliated fibroblasts. The number was significantly reduced in FA.II-1, FA.II-2 and FB.II-2 (p<0.0001) compared to the controls (C) (two controls were used and pooled for the calculation). The numbers of the cells analyzed are indicated on top of the bars.

(b) Staining for ARL13B and glutamylated Tubulin (GT335) detected a significant increase in ciliary length in the affected individuals FA.II-1, FA.II-2 and FB.II-2 compared to controls. Median ciliary length difference between affected individuals and control cells were 1.1 µm, 1.4 µm and 2.1 µm respectively (p<0.0001).

Statistical analysis of the immunofluorescence data was performed using ggplot2 (https://ggplot2.tidyverse.org/), data were analyzed by unpaired t-test with Welch's correction error bars representing the standard error of the mean (s.e.m.).

**
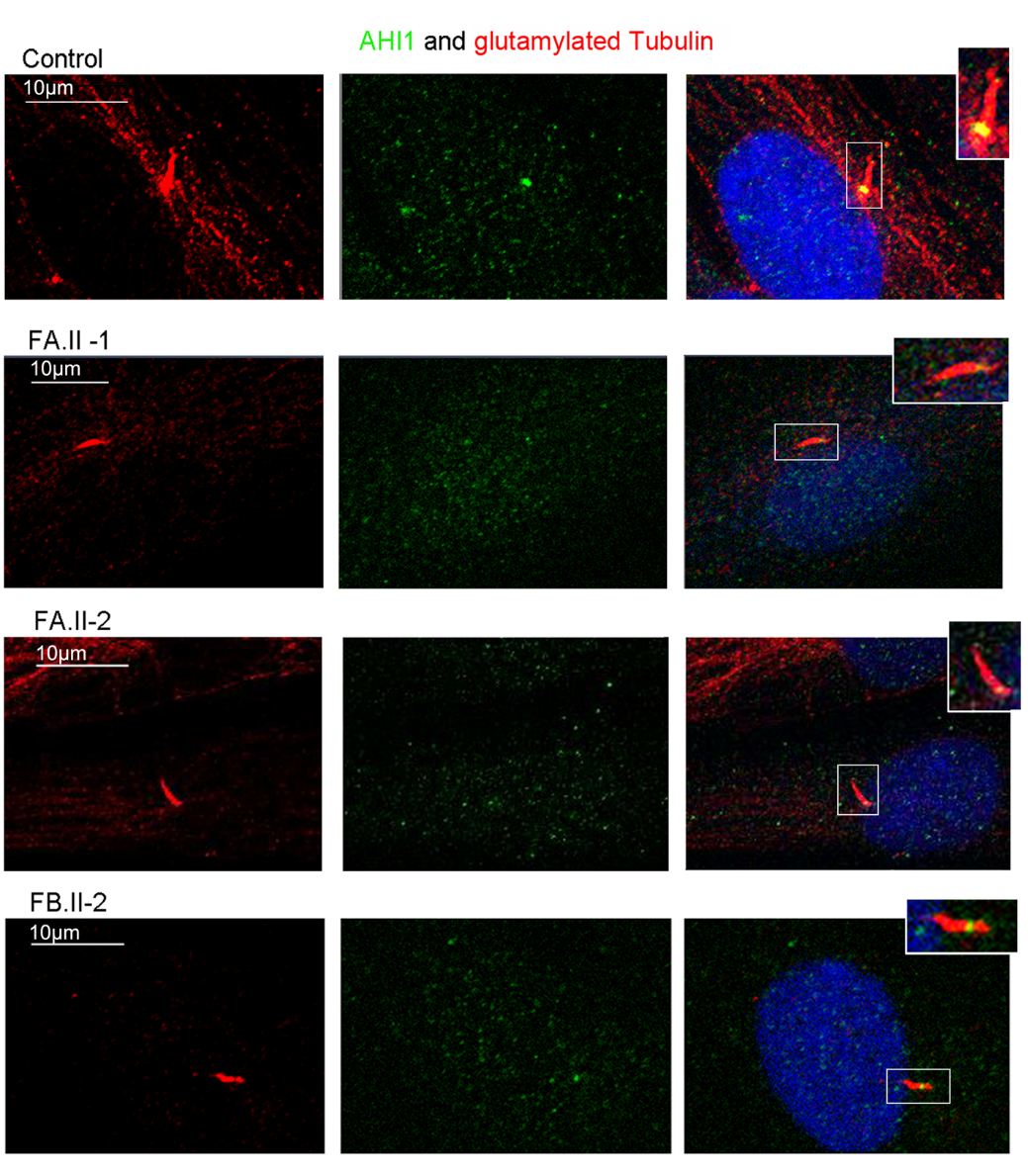
**

**Figure S6 Immunofluorescence study of fibroblasts showed reduced AHI1 signals in cells from FA.II-1, FA.II-2 and FB.II-2 compared to control cells.**

Fibroblasts were stained for AHI1 (Alexa 488, green) and glutamylated Tubulin (GT335) (Alexa 568, red) used as ciliary markers. Cells from FA.II-1, FA.II-2 and FB.II-2 showed reduced AHI1 staining compared to controls. The white squares indicate the region of interest, which are shown as magnified inserts.


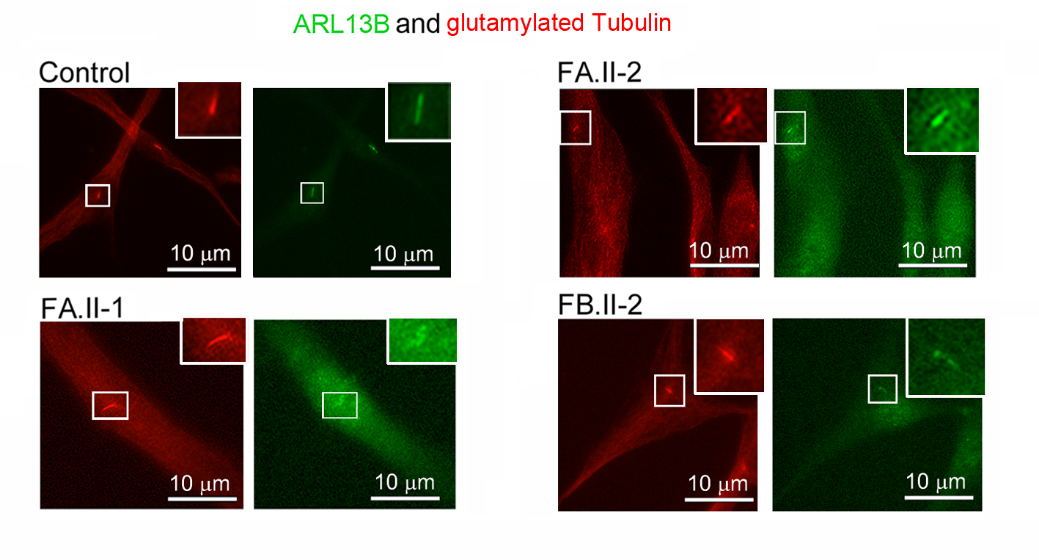


**Figure S7 Immunofluorescence study of fibroblasts showed reduced ARL13B signals in cells from FA.II-1, FA.II-2 and FB.II-2 compared to control cells.**

Fibroblasts were stained for ARL13B (Alexa 488, green) and glutamylated Tubulin (GT335) (Alexa 568, red) used as ciliary markers. Cells from FA.II-1, FA.II-2 and FB.II-2 showed reduced ARL13B staining compared to controls. The white squares indicate the region of interest, which are shown as magnified inserts.


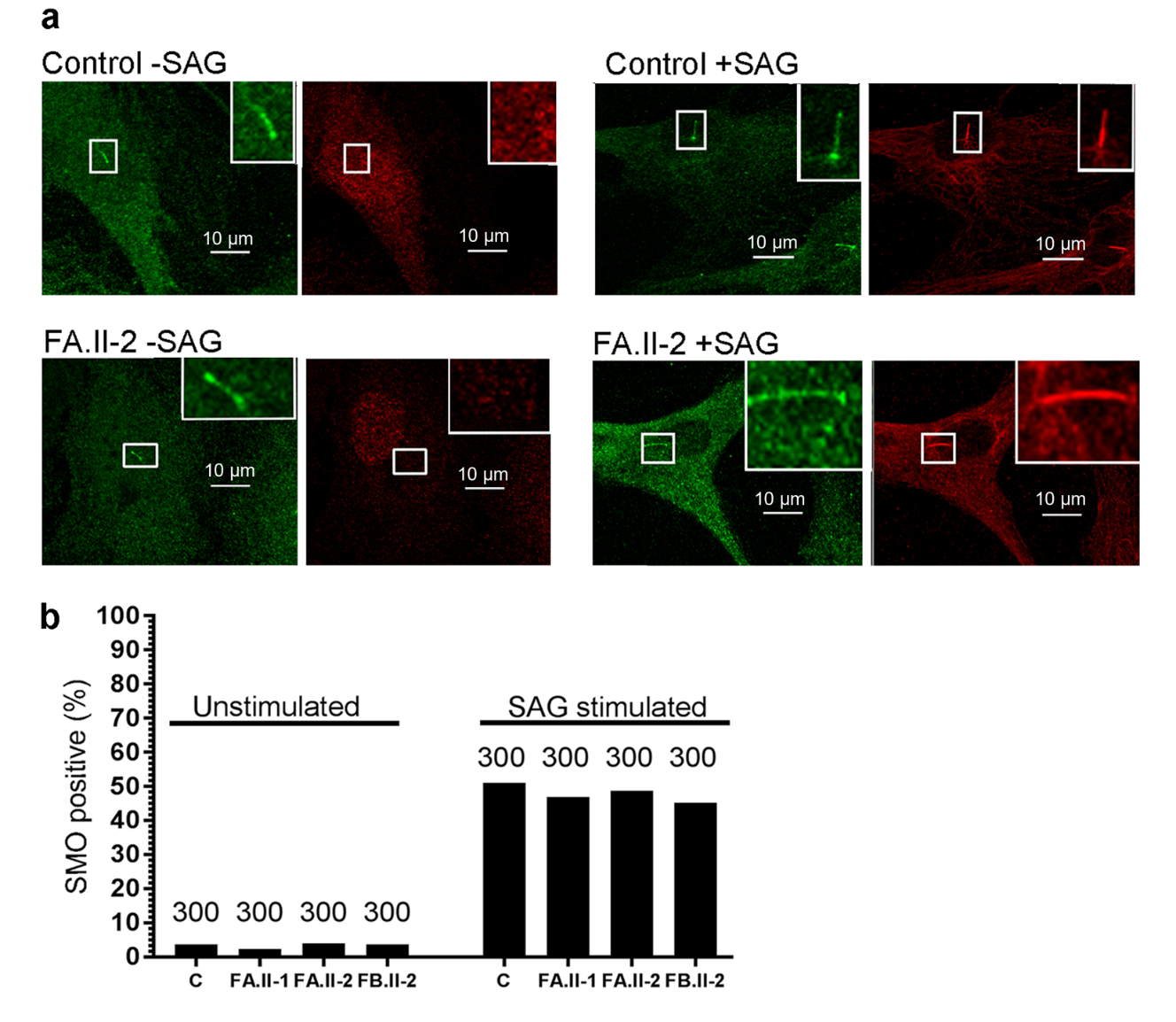


**Figure S8 SMO recruitment to the ciliary membrane upon SAG stimulation.**

(a) Ciliated fibroblasts from one control and affected individuals FA.II-1, FA.II-2 and FB.II-2 after 72 hours serum starvation (representative images from FA.II-2 are shown). One set of cells was treated with SAG for 24 hours (+SAG) and the other set of cells was kept untreated for 24 hours (-SAG). Cells were stained with IFT88 (Alexa488, green) to identify the ciliary axoneme. Cells positive for IFT88 and also positive for SMO (Cy3, red) in the ciliary membrane were counted. Note the SMO recruitment into ciliary membrane upon SAG stimulation. The inserts indicate the regions of interest, which are shown as magnified in one corner.

(b) SMO was recruited into the ciliary membrane in 45-51% of the cilia when stimulated with SAG whereas only 2-4% of the cilia were positive for SMO signal in unstimulated cells. The results show no significant differences in ciliary SMO recruitment in the three affected individuals compared to the controls. The numbers of cells counted per treatment group, per individual are reported above each bar.


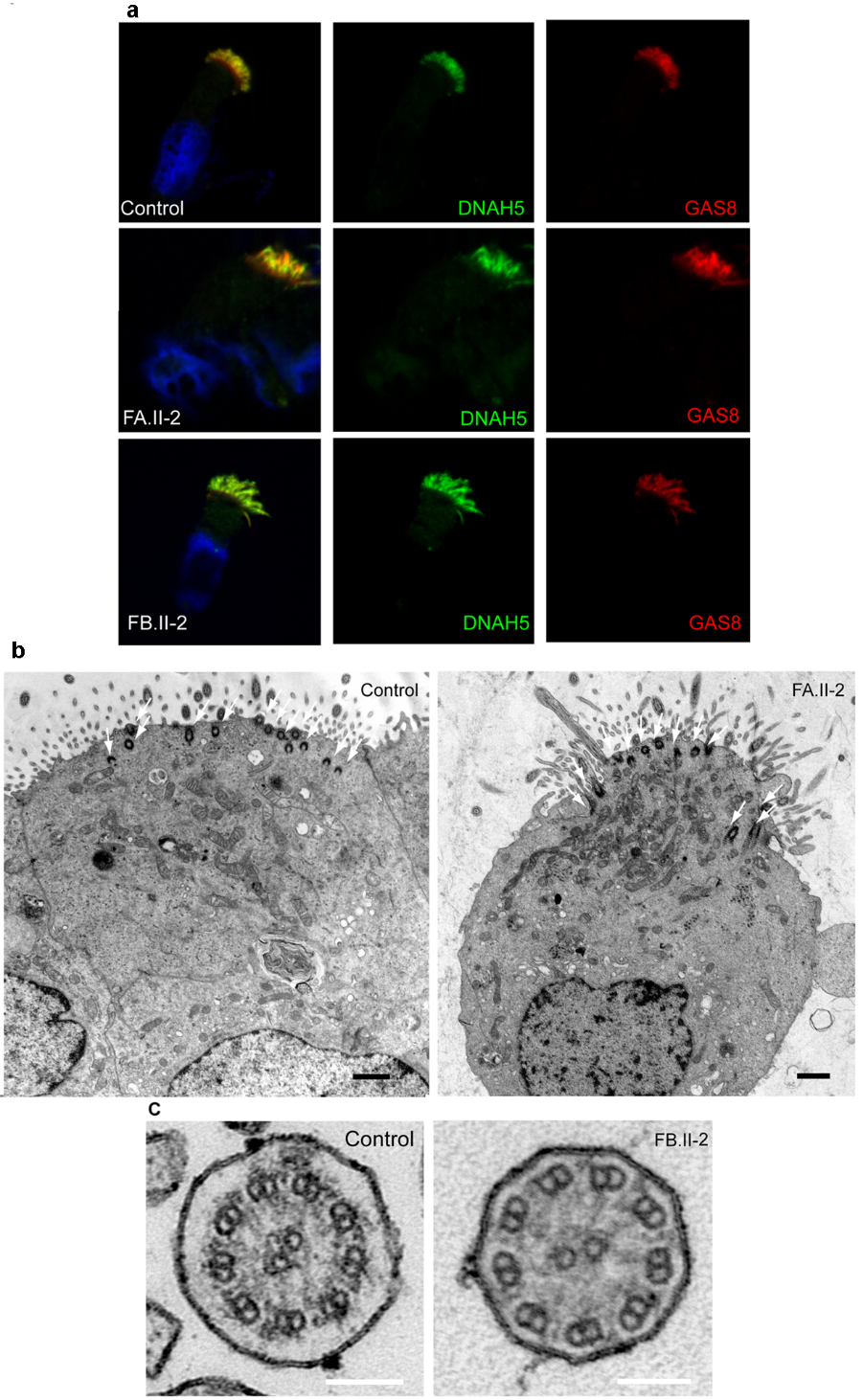


**Figure S9 Characterization of motile cilia in respiratory epithelial cells obtained by nasal brush biopsy.**

(a) Staining for the outer dynein arm protein DNAH5 (Alexa 488, green) and the nexin-dynein regulatory complex protein GAS8 (Alexa 546, red) which co-localize within the ciliary axonemes revealed no differences in respiratory epithelial cells from FA.II-2 and FB.II-2 compared to the control. Scale bars correspond to 10 µm.

(b) Transmission electron microscopy (TEM) analyses of motile cilia in respiratory epithelial cells from FA.II-2 and FB.II-2 did not reveal reduced numbers of basal bodies, but a few basal bodies were mislocalized within the cytoplasm and not docked to the plasma membrane (indicated with white arrows). Scale bars 1 µm.

(c) TEM analyses revealed normal ciliary ultrastructure in CBY1 deficient respiratory epithelial cells from FA.II-2 and FB.II-2. Scale bars 200 nm.

Methods details: Respiratory epithelial cells obtained by nasal brush biopsy were suspended in cell culture medium. Samples were spread onto glass slides, air-dried and stored at -80°C until use. Spheroid cultures from nasal brushings were performed as reported (Olbrich et al., 2006). Cells were treated with 4% paraformaldehyde (or 100% ice-cold methanol) and 0.2% Triton X-100. The slides used for anti-GAS8/DNAH5 staining were blocked overnight with 1% skim milk before incubation with the primary antibodies for about 3 to 4 hours at room temperature the next day. The incubation with the secondary antibodies was performed for 30 minutes at room temperature. Rabbit polyclonal antibody directed against DNAH5 has been reported (Fliegauf et al., 2005). Rabbit polyclonal anti-GAS8 (1:500) was obtained from Atlas Antibodies AB, Bromma, Sweden. The primary and secondary antibodies are listed in Supporting information Table S2.

Transmission electron microscopy (TEM). Nasal biopsies were taken from the middle turbinate. The samples were fixed in 2.5% glutaraldehyde and washed with 1.3% osmium tetroxide. The samples were embedded in 1,2-epoxypropan-epon mixture (1:1) at 4°C overnight. After polymerization, several sections were picked out onto copper grids. The sections were stained with Reynold’s lead citrate. TEM was performed with the Philips CM10 (Philips, Amsterdam, Netherlands).

HVMA was carried out directly after brushing. Videos were recorded using a Basler scA640-120 fm digital high-speed video camera (Basler AG, Ahrensburg, Germany) attached to an inverted phase-contrast microscope (Zeiss Axio Vert. A1; Carl Zeiss AG, Göttingen, Germany) equipped with a 40× objective. Image processing was performed using (Sisson-Ammons Video Analysis) SAVA (Sisson, Stoner, Ammons, & Wyatt, 2003). The temperature was maintained at 25°C by a Pecon Temp Controller 2000-2 (PeCon GmbH, Erbach, Germany). HVMA in FA.II-2 and FB.II-2 revealed beating frequency in the low-normal range (4-8 Hz, reference range 4-12 Hz at 26 °C) (Raidt et al., 2014).

The ciliary length was normal.

**References**

Fliegauf, M., Olbrich, H., Horvath, J., Wildhaber, J. H., Zariwala, M. A., Kennedy, M., . . . Omran, H. (2005). Mislocalization of DNAH5 and DNAH9 in respiratory cells from patients with primary ciliary dyskinesia. *Am J Respir Crit Care Med, 171*(12), 1343-1349. doi:10.1164/rccm.200411-1583OC

Olbrich, H., Horvath, J., Fekete, A., Loges, N. T., Storm van's Gravesande, K., Blum, A., . . . Omran, H. (2006). Axonemal localization of the dynein component DNAH5 is not altered in secondary ciliary dyskinesia. *Pediatr Res, 59*(3), 418-422. doi:10.1203/01.pdr.0000200809.21364.e2

Raidt, J., Wallmeier, J., Hjeij, R., Onnebrink, J. G., Pennekamp, P., Loges, N. T., . . . Werner, C. (2014). Ciliary beat pattern and frequency in genetic variants of primary ciliary dyskinesia. *Eur Respir J., 44*(6), 1579-1588. doi:10.1183/09031936.00052014

Sisson, J. H., Stoner, J. A., Ammons, B. A., & Wyatt, T. A. (2003). All-digital image capture and whole-field analysis of ciliary beat frequency. *J Microsc, 211*(Pt 2), 103-111.

**Table S1 Additional clinical features in Family A**

| **Features** | **II-1** | **II-2** | **II-3** | **II-4** |
| --- | --- | --- | --- | --- |
| Age at last examination (years) | 21 y | 18 y | 13 y | 10 y |
| IQ assessment | 64 | 63 | 69 | Normal school performance |
| Microcephaly | + | - | +^a^ | + |
| Neuromuscular disease | +^b^ | - | - | +^b^ |
| Hypogonadism | +^c^ | - | +^c, d^ | +^d^ |
| Nasal septum deviation | + | + | - | +^e^ |
| Bilateral cataracts (operated) | + | - | + | + |
| Recurrent kerato-conjunctivitis | - | + | - | - |
| Recurrent painful sub-ungual hemorrhage in all digits | + | - | - | - |
| Coeliac disease | + | - | - | - |
| Headache | - | + | - | - |

Legend. ^a^ OFC 49 cm (2 cm <2.5^th^ percentile) at 13.5 years.

^b^ Muscle pain and weakness and elevated CK. II-1: CK 350 to 1200 U/L; II-4: CK 305-460 U/L (reference < 200 U/L). MRI showed fatty infiltration in muscle tissue.

Neurophysiological examination showed motor and sensory neuropathy.

Histology of the muscle biopsy indicated extensive myopathy and neurogenic changes in II-1 and neurogenic changes in II-4.

^c^ II-1 Testosterone 7.9 mmol/L (reference 7.2-24), FSH 18.4 IU/L (reference 0.7-11.1), LH 12.5 IU/L (reference 0.8-7.6). Testicular volume was 3-4 ml on each side on ultrasound (corresponding to pre-pubertal testicular size).

II-4 P-Estradiol < 0.06 nmol/L (reference 0.09-0.21), P-FSH >170 IU/L (reference 2.8-11.3) and P-LH 90.4 IU/L (reference 1.1-11.6), suggesting primary ovarian insufficiency.

^d^ Pelvis MRI revealed agenesis/hypoplasia of the ovaries and the uterus.

^e^ Awaiting operation.

| **Table S2 Antibodies used in IF studies in fibroblasts and respiratory epithelial cells** | | | | | |
| --- | --- | --- | --- | --- | --- |
| **Primary antibody** | **Host** | **Provider** | **Catalog number** | **Dilution** | **Fixation method** |
| anti-acetylated Tubulin monoclonal | Mouse | Sigma-Aldrich, St. Louis, MO, USA | T7451 | 1:500  1:10000 | 100% Methanol  4% Formaldehyde +  0,2% Triton-X |
| anti-AHI1 polyclonal | Rabbit | Proteintech, Rosemont, IL, USA | 22045-1-AP | 1:400 | 100% Methanol |
| anti-ARL13B polyclonal | Rabbit | Proteintech | 17711-1-AP | 1:1000 | 100% Methanol |
| anti-CEP164 polyclonal | Rabbit | Sigma-Aldrich | HPA037606 | 1:1000 | 100% Methanol |
| anti-Chibby monoclonal | Mouse | Santa Cruz biotechnology  Dallas, TX, US | SC-101551 | 1:200 | 100% Methanol |
| anti-DNAH5 | Rabbit | From Fliegauf 2005 |  | 1:1000 | 4% Formaldehyde +  0,2% Triton-X |
| anti-GAS8 polyclonal | Rabbit | Atlas Antibodies | HPA041311 | 1:500 | 4% Formaldehyde +  0,2% Triton-X |
| polyglutamylated Tubulin mAb GT335 polyclonal | Mouse | Adipogen, San Diego, CA, USA | AG-20B-0020 | 1:1000 | 100% Methanol or 100% Methanol +  4% Formaldehyde |
| anti-IFT88 polyclonal | Rabbit | Proteintech | 13967-1-AP | 1:500 | 100% Methanol +  4% Formaldehyde |
| anti-SMO | Mouse | Santa Cruz biotechnology  Dallas, TX, US | sc-166685 | 1:200 | 100% Methanol +  4% Formaldehyde |
| **Secondary antibodies** | **Host** | **Provider** | **Catalog number** | **Dilution** |  |
| Cy3 conjugated AffiniPure  Donkey Anti-Mouse IgG | Mouse | Jackson ImmunoResearch Laboratories, Suffolk, UK | 715-165-150 | 1:1000 |  |
| Alexa Fluoro-488 conjugated AffiniPure  Donkey Anti-Rabbit IgG | Rabbit | Jackson ImmunoResearch Laboratories | 711-545-152 | 1:1000 |  |
| Alexa Fluor 488 Goat Anti-Mouse IgG (H+L) | Mouse | ThermoFisher scientific, MA, USA | A11029 | 1:1000 |  |
| Alexa Fluor 546 Goat Anti-Rabbit IgG (H+L) | Rabbit | ThermoFisher scientific | A11035 | 1:1000 |  |

Legend. List of antibodies used in IF studies in fibroblasts and respiratory epithelial cells.

**Table S3 WES coverage and analyses in Family A**

| **Panel A. WES coverage in Family A** | | | | | |
| --- | --- | --- | --- | --- | --- |
| **Individual** | **FA.I-1** | **FA.I-2** | **FA.II-1** | **FA.II-2** | **FA.II-3** |
| Capture kit Agilent_SureSelect_AllExon | v06r2 | v05 | v05 | v05 | v06r2 |
| Reads | \| 179763886 \| \| --- \| | 62791080 | 60424508 | 201102176 | 91675886 |
| Fraction of unique reads aligning to reference | 0.997 | 0.976 | 0.976 | 0.973 | 0.960 |
| Mean target coverage | 159.2 | 72.1 | 72.1 | 216.3 | 113.8 |
| Fraction of bases in target covered >10x | 0.977 | 0.978 | 0.975 | 0.989 | 0.971 |
| Fraction of bases in target covered >20x | 0.971 | 0.938 | 0.927 | 0.984 | 0.958 |

| **Panel B. Recessive variants common to FA.II-1 and FA.II-2** | | | | | | |
| --- | --- | --- | --- | --- | --- | --- |
| **Gene** | **MIM** | **Genomic annotation (GRCh37)** | **gnomAD** | **CADD** | **SIFT** | **Poly-Phen2** |
| *ABCA13* | NA | 7:48273747A>T;E299V | 6.02E-5 | 23.7 | \| 0 \|  \| \| --- \| --- \| | 0.996 |
|  |  | 7:48413946C>G;C3712W | 1.912E-4 | 24.4 | 0 | 0.995 |
| *CARD10* | NA | 22:37912050G>A;A210V | 7.428E-5 | 32.0 | \| 0 \|  \| \| --- \| --- \| | 0.994 |
| *CBY1* | NA | 22:39067079_39067080delAG | 3.579E-5 | 34.0 |  |  |
| *HERC1* | Macrocephaly, dysmorphic facies, and psychomotor retardation, AR, MIM# 617011 | 15:63967239G>A | 7.226E-4 | 19.8 | 0.11 | 0 |
|  |  | 15:64005777C>T;R1413Q | 6.564E-4 | 22.7 | 0.58 | 0.198 |
| *SCUBE1* | NA | 22:43619184G>A;R416W | 3.33E-5 | 22.6 | 0.11 | 0 |
| *TRPM1* | Night blindness, congenital stationary (complete), 1C, AR, MIM# [613216](https://www.omim.org/entry/613216) | 15:31318408T>A;H1205L | 1.134E-3 | 25.2 | 0.02 | 0.021 |
|  |  | 15:31329943C>T;A865T | 5.338E-5 | 28.5 | 0.01 | 0.998 |

| **Panel C. Recessive variants present in FA.II-1 that are not present in the parents and the siblings FA.II-2 and FA.II-3** | | | | | | |
| --- | --- | --- | --- | --- | --- | --- |
| **Gene** | **MIM** | **Genomic annotation** | **gnomAD** | **CADD** | **SIFT** | **Poly-Phen2** |
| *FCGBP* | NA | 19:40363221C>T;R4950Q | 2.19E-04 | 22.9 | 0.61 | 0.752 |
|  |  | 19:40366565C>T;G4557S | 1.99E-03 | 28.1 | 0.03 | 0.983 |
| *ATP1B4* | NA | X:119513337A>T;T265S | 3.27E-03 | 20.1 | 0.22 | 0.41 |
| *FBXW10* | NA | 17:18659398C>T;T388M | 7.69E-04 | 25.5 | 0 | 0.959 |
| *GOLGB1* | NA | 3:121412618T>C;H2251R | 6.98E-03 | 16.95 | 0.29 | 0.127 |
| *HIPK3* | NA | 11:33308763G>A;R268H | 8.14E-05 | 24.3 | 0.02 | 0.667 |
| *KRT13* | White sponge nevus 2, AD, MIM# 615785 | 17:39659217C>T;R290Q | 3.25E-03 | 23.4 | 0.03 | 0.313 |
| *MYO1D* | NA | 17:30980871T>A;H862L | 2.54E-03 | 21.5 | 0.34 | 0.186 |
| *PCYT1B* | NA | X:24690692G>A;R20C | 1.37E-04 | 24.9 | 0 | 0.673 |
| *PLSCR5* | NA | 3:146322998A>G (start codon loss) | 2.66E-03 | 23.3 | 0 | 0.777 |
| *RSAD1* | NA | 17:48557249CTG>C | 1.72E-04 | 34 |  |  |
| *RTN4IP1* | Optic atrophy 10 with or without ataxia, mental retardation, and seizures, AR, MIM# 616732 | 6:107035582C>G;G321A | 7.12E-04 | 23 | 0.51 | 0.144 |
| *SLFN14* | Bleeding disorder, platelet-type, 20, AD, MIM 616913 | 17:33884703G>A (intronic variant) | 4.58E-04 | 17.56 | 0.16 | 0.65 |
| *SYCP2* | NA | 20:58455447A>G;I951T | 4.61E-03 | 25.3 | 0 | 0.523 |
| *THRA** | Hypothyroidism, congenital, nongoitrous, 6, AD, MIM # 614450 | 17:38243095A>C;M238L | 3.66E-05 | 22.6 | 0.28 | 0.021 |
| *TPRA1* | NA | 3:127294793A>C;F200C | 2.07E-04 | 22.2 | 0.07 | 0.014 |
| *UTP18* | NA | 17:49343560A>G;N158S | 6.67E-04 | 18.79 | 0.08 | 0.175 |

* FA.II-1 was tested four times for thyroid status and had normal values for FT3, FT4 and TSH.

| **Panel D. Recessive variants present in FA.II-1 and FA.II-3 that are not present in the mother and the siblings FA.II-2** | | | | | | |
| --- | --- | --- | --- | --- | --- | --- |
| **Gene** | **MIM** | **Genomic annotation** | **gnomAD** | **CADD** | **SIFT** | **Poly-Phen2** |
| *NHP2* | Dyskeratosis congenita, autosomal recessive 2, AR, MIM*#*613987 | 5:177580704C>T;A39T | 4.27-05 | 31 | 0 | 0.945 |
| *TMEM132A* | NA | 11:60696359G>A;V265M | 2.08E-04 | 21 | 0.02 | 0.132 |
| *HTT* | Huntington disease, AD, MIM # 143100  Lopes-Maciel-Rodan syndrome, AR, MIM 617435 | 4:3076603CCAGCAG>C,CCAGCAGCAGCAG (inframe insertion) | 3.67E-02 | 18 |  |  |
| *LRRC10B* | NA | 11:61277197C>T; P243S | 1.59E-04 | 23 | 0.09 | 0.992 |

Legend. CADD Combined Annotation Dependent Depletion score (the scaled C-score) was used; NA, not available.

**Table S4 Cohort of individuals with a clinical diagnosis of ciliopathy reanalyzed for biallelic variants in *CBY1***

| **Screening centers** | **Clinical diagnosis** | **Individuals without genetic diagnosis** | **Total number of individuals** |
| --- | --- | --- | --- |
| International Radboud Institute for Molecular Life Sciences, Radboud University Nijmegen, NL | BBS | 4 | 42 |
|  | SRTD | 13 | 64 |
|  | Laterality defects  (none with PCS) | 26 | 42 |
|  | Sensenbrenner/CED | 6 | 6 |
| Department of Pediatrics, University of Washington and Seattle Children's Research Institute, Seattle, WA | JBTS, MKS | 215 | 544 |
| Department Of Nephrology and Hypertension, University Medical Center Utrecht, Heidelberglaan 100, 3584CX, Utrecht, NL | NPHP | 105 | 105 |
| Center for Human Disease Modeling, Duke University Medical Center, Durham, NC | BBS, JATD, MKS, PCD, OFD, USH | 269 | 457 |
| **TOTAL** |  | **638** | **1260** |

Legend. BBS, Bardet-Biedl syndrome; CED, Cranioectodermal dysplasia; JATD, Jeune asphyxiating thoracic dystrophy; JBTS, Joubert syndrome; MKS, Meckel syndrome; NPHP, Nephronophthisis; OFD, Orofaciodigital syndrome; PCD, Primary ciliary dyskinesia; SRTD, Short rib thoracic dysplasia syndrome; USH, Usher syndrome.
